# Violence risk assessment instruments in forensic psychiatric populations: a systematic review and meta-analysis

**DOI:** 10.1016/S2215-0366(23)00256-0

**Published:** 2023-10

**Authors:** Maya G T Ogonah, Aida Seyedsalehi, Daniel Whiting, Seena Fazel

**Affiliations:** a Department of Psychiatry, University of Oxford, Warneford Hospital, Oxford, UK; b Institute of Mental Health, University of Nottingham, Nottingham, UK

## Abstract

**Background:**

Although structured tools have been widely used to predict violence risk in specialist mental health settings, there is uncertainty about the extent and quality of evidence of their predictive performance. We aimed to systematically review the predictive performance of tools used to assess violence risk in forensic mental health, where they are routinely administered.

**Methods:**

In our systematic review and meta-analysis, we followed PRISMA guidelines and searched four databases (PsycINFO, Embase, Medline, and Global Health) from database inception to Nov 1, 2022, to identify studies examining the predictive performance of risk assessment tools in people discharged from forensic (secure) mental health hospitals. Systematic and narrative reviews were excluded from the review. Performance measures and descriptive statistics were extracted from published reports. A quality assessment was performed for each study using the Prediction Model Risk of Bias Assessment Tool. Meta-analysis was conducted on the performance of instruments that were independently externally validated with a sample size greater than 100. The study was registered with PROSPERO, CRD42022304716.

**Findings:**

We conducted a systematic review of 50 eligible publications, assessing the predictive performance of 36 tools, providing data for 10 460 participants (88% men, 12% women; median age [from 47 studies] was 35 years, IQR 33–38) from 12 different countries. Post-discharge interpersonal violence and crime was most often measured by new criminal offences or recidivism (47 [94%] of 50 studies); only three studies used informant or self-report data on physical aggression or violent behaviour. Overall, the predictive performance of risk assessment tools was mixed. Most studies reported one discrimination metric, the area under the receiver operating characteristic curve (AUC); other key performance measures such as calibration, sensitivity, and specificity were not presented. Most studies had a high risk of bias (49 [98%] of 50), partly due to poor analytical approaches. A meta-analysis was conducted for violent recidivism on 29 independent external validations from 19 studies with at least 100 patients. Pooled AUCs for predicting violent outcomes ranged from 0·72 (0·65–0·79; *I*^2^=0%) for H10, to 0·69 for the Historical Clinical Risk Management-20 version 2 (95% CI 0·65–0·72; *I*^2^=0%) and Violence Risk Appraisal Guide (0·63–0·75; *I*^2^=0%), to 0·64 for the Static-99 (0·53–0·73; *I*^2^=45%).

**Interpretation:**

Current violence risk assessment tools in forensic mental health have mixed evidence of predictive performance. Forensic mental health services should review their use of current risk assessment tools and consider implementing those with higher-quality evidence in support.

**Funding:**

Wellcome Trust.

## Introduction

Violence perpetrated by individuals after hospital discharge from forensic psychiatric care is a major concern for psychiatric services, with costs to the wider economy, public health, and social care.^1^ Such individuals continue to present a risk of violence following discharge, with a meta-analysis^2^ finding crude violent reoffending rates of 3902 per 100 000 person-years, equivalent to approximately 4% per year. Estimates for reconviction rates for violent offences stand at approximately 12% within 2 years of discharge.^3^ Those who enter forensic psychiatric services often do so as an alternative to prison, with an expectation that access to specialist mental health treatment will more effectively reduce reoffending risk.

Assessing and managing post-discharge violence risk is a core feature of forensic psychiatric care; risk assessment instruments aid in the identification of individuals at risk of violence perpetration and assist in risk management. Owing to the potential use of such tools, and with increasing recognition of the importance of predicting violence perpetration, violence risk assessment has been the subject of much clinical and research interest. More than 400 risk assessment instruments designed to assess the risk of violence and offending have been developed,^4^ and many have been implemented in forensic mental health around the world. Despite their widespread use, definitive syntheses of evidence for their predictive performance are scarce. Previous reviews are outdated^5^, ^6^, ^7^ or do not follow reporting guidelines, and uncertainties remain regarding the use of risk assessment instruments in new settings and populations. Furthermore, previous reviews typically combine inpatient violence with post-release community violence.^5^, ^7^, ^8^ As violence base rates, key risk factors, and the type of intervention differ between inpatient samples and individuals in the community, a review that disaggregates inpatient and post-discharge samples is necessary. Previous research^9^ has sought to review the performance of risk assessment instruments in inpatient samples; however, to our knowledge, there are no syntheses specifically assessing tool performance in predicting post-release violence.

**Research in context**

**Evidence before this study**

Violence perpetrated by individuals after discharge from hospital for forensic psychiatric care is a major concern for psychiatric services. Although various risk assessment tools have been developed to assist with risk prediction and management, it is not known which tools are supported by high quality evidence and which are most accurate. We searched PubMed from database inception to Jan 10, 2022, without language restrictions using the search term (risk assess*) AND (predict* OR accura* OR “psychometric properties”) AND (violen* OR crime) AND (“systematic review” OR “Meta-analy*”) as a filter. We identified five relevant systematic reviews or meta-analyses that examined one or a limited number of tools, combined populations (eg, from both prison and forensic settings), combined inpatient and community outcomes, or only examined inpatient violence. We found no systematic reviews or meta-analyses specifically examining the predictive performance of risk assessment tools in forensic psychiatric patients after discharge. To develop guidance for clinicians and policy makers on which risk assessment instruments should be considered in forensic mental health, clarification of the predictive performance of individual instruments is necessary.

**Added value of this study**

This comprehensive synthesis of the performance of 36 risk assessment instruments used in forensic mental health settings in 10 460 participants found that most research on these tools is at high risk of bias and has only reported one performance measure—the area under the receiver operating characteristic curve (AUC)—rather than a basic suite of tests of discrimination (sensitivity and specificity) and calibration. Estimates of the pooled AUC for predicting violent recidivism ranged from 0·64 to 0·72. Furthermore, there was little research on women in forensic psychiatric populations (only two studies assessed risk assessment tools in female-only samples) and there was no research in low-income or middle-income countries.

**Implications of all the available evidence**

In forensic mental health services, the implementation of high quality risk assessment tools can complement clinical decision making. However, these tools should not be used to inform decisions of lengths of stay without validations showing high sensitivity, and they should only be used to inform discharge planning and resource allocation when validations show high levels of specificity. Benchmarks for acceptable sensitivity and specificity need clarification. This meta-analysis suggests current practice needs review. In the case of new services, including in low-income and middle-income countries, risk assessment tools that are introduced should have validation studies in support, with information on discrimination (including rates of false positives and negatives) and calibration provided.

Despite its importance, very few instruments have been designed for violence risk assessment in forensic psychiatric patients after hospital discharge. Current clinical guidelines recommend using structured assessment tools, such as the Psychopathy Checklist—Revised (PCL-R), Psychopathy Checklist—Screening Version (PCL-SV), and the (HCR-20), for use in forensic populations, which can also assist in incorporating personality difficulties into risk assessment.^10^ US guidelines cite risk assessment tools as a useful memory aid, but do not recommend specific tools.^11^ Similarly, European guidelines recommend the use of structured professional judgement risk assessment instruments in forensic psychiatry, but do not suggest specific tools.^12^ To develop such guidance, research synthesis that examines the performance of individual instruments is necessary.

Previous reviews assessing risk assessment tools in psychiatry have tended to include mostly men.^2^, ^5^, ^10^ Women constitute approximately 5–18% of forensic psychiatric patients in Europe,^13^, ^14^ and have differential pathways to crime and forensic services compared with men, including risk, markers such as childhood victimisation,^15^, ^16^ emotional and cognitive dysfunction,^17^ relational difficulties, and intimate partner violence.^18^ Thus, it is important to know whether commonly used risk assessment instruments are applicable to women.

This study aims to systematically review and meta-analyse the performance of risk assessment instruments used to predict interpersonal violence and crime in forensic psychiatric patient samples after discharge.

## Methods

### Search strategy and selection criteria

We report our findings according to the Preferred Reporting Items for Systematic Reviews and Meta-Analyses (PRISMA; appendix pp 11–14).^19^ Based on recent systematic reviews and international surveys,^4^, ^20^, ^21^, ^22^, ^23^ the 15 most commonly used violence risk prediction instruments in forensic psychiatric samples were identified to inform the search strategy, although the search was not limited to these 15 instruments.

A systematic search was conducted to identify studies that measured the performance of risk assessment instruments in predicting the outcome of interpersonal violence and crime in forensic psychiatric samples post-discharge. Four databases (PsycINFO, Embase, Medline, and Global Health) were searched separately from their start date until Nov 1, 2022. No limits, restrictions (including by language), or published search filters were used (appendix p 15). The first 100 results on Google Scholar and the reference list of previous systematic reviews were browsed to try and identify additional studies.

We included studies of forensic psychiatric patients or psychiatric patients admitted to secure units following violent or criminal incidents, and studies that assessed the predictive performance of a risk assessment instrument at predicting post-discharge interpersonal violence and crime using at least one commonly accepted performance metric (sensitivity, specificity, positive predictive value, negative predictive value, c-index or area under the receiver operating characteristic curve [AUC], and calibration). Both retrospective cohort and prospective cohort studies were included. Systematic, narrative, and book reviews were excluded from the review. Non-nested case-control studies were excluded as they cannot be used to estimate absolute risk, leading to incorrect estimates of baseline hazard. Sexual offenders referred for civil commitment were excluded from this review (appendix p 2).

MGTO screened the titles and abstracts of all identified studies, with 10% double screened by reviewer AS to ensure adequate interrater reliability. Cohen's κ was used to calculate inter-rater agreement,^24^ which was 0·95, indicating almost perfect agreement between the two raters.^25^ Any disagreements were resolved via consensus. Reference lists of retained studies were hand searched to identify additional studies.

In the prespecified study protocol, the main outcome was defined using the umbrella term interpersonal violence and crime. However, to be consistent with how the outcome was defined in the papers reviewed, we modified it to recidivism, subcategorised into violent, general, and sexual recidivism.

### Data analysis

One reviewer (MGTO) extracted study characteristics and summary estimates and a second reviewer (AS) independently verified a random 10% subset of full-text articles; any uncertainties were referred to the senior author (SF). Disagreements were resolved via consensus. Individual studies could report on more than one risk assessment instrument, so we extracted information on each instrument. Studies often reported multiple types of interpersonal violence or crime (eg, violent and general recidivism), so all outcome measures were extracted from each paper. If multiple publications were identified corresponding to a single study, the most complete report was chosen for data extraction and supplemented using data from associated publications. Authors were contacted when insufficient information was in the publication.

Following Cochrane guidelines,^26^, ^27^ a meta-analysis of the predictive performance of externally validated risk assessment instruments was conducted (appendix p 3). When a tool had been validated at least three times for the outcome, we applied a random effects model, using the inverse-variance method, for pooling the logit transformation of the AUC and CIs. The predictive performance of each risk assessment instrument was pooled across all external validation studies regardless of study design (eg, including both retrospective and prospective cohort studies), as recommended for meta-analyses of prediction model studies.^28^ To reduce bias, only independent validation studies with a sample size that is consistent with adequate statistical power were included in the primary analysis. We set this threshold at n=100, as a balance between the current methodological recommendations for minimum event numbers for validation studies^29^ and excluding too large a proportion of existing literature. As a secondary analysis, we provide a narrative summary of the predictive performance of all studies, irrespective of authorship, validation, or sample size, and a post-hoc subgroup of analysis of AUCs by prediction horizon (categorised as up to 12 months, 1–5 years, and more than 5 years). Analyses were performed with R version 4.1.0 using the metafor^30^ and meta-package^31^, ^32^ (appendix p 3).

The Prediction Model Risk of Bias Assessment Tool (PROBAST),^33^ designed to provide guidance on the quality assessment for systematic reviews of studies investigating diagnostic and prognostic prediction models, was adapted and provided a risk of bias rating for each study, with low, high, or unclear risk of bias categorisations (appendix p 3). The tool consists of four domains (participants, predictors, outcomes, and analysis) containing 20 signalling questions to facilitate a risk of bias assessment. A domain where all signalling questions are answered as yes or probably yes is judged as having a low risk of bias. Any answers of no or probably no for one or more questions results in a high risk of bias in that domain. The overall risk of bias is judged as high if at least one domain is rated as being at high risk of bias. MGTO assessed the risk of bias for each included study based on PROBAST guidelines, with 10% re-rated by reviewer AS. Any disagreements were resolved by consensus.

This study was registered with PROSPERO, CRD42022304716.

### Role of the funding source

The funder had no role in study design, data collection, data analysis, data interpretation, or writing of the report.

## Results

**Figure 1:**
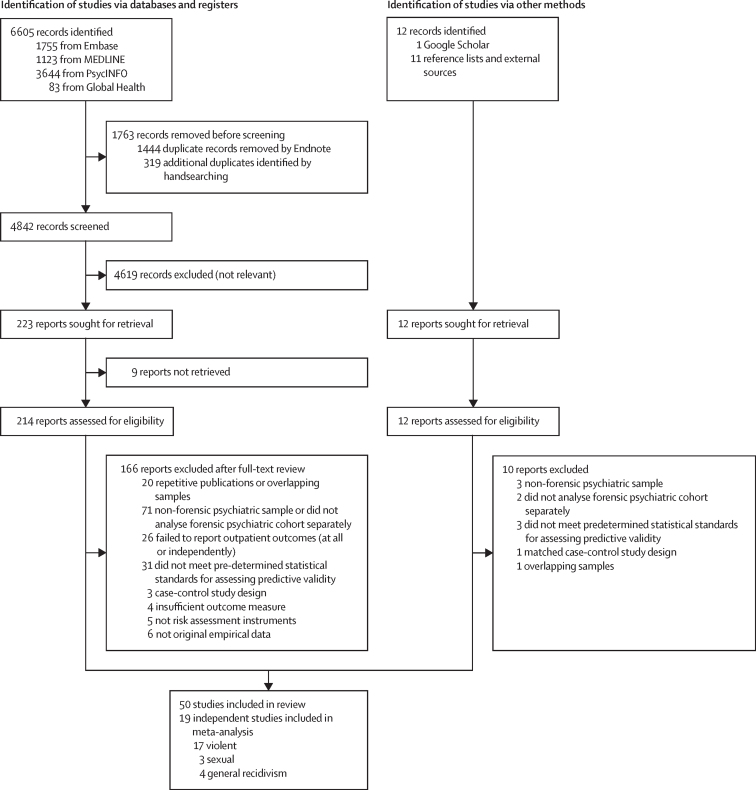
PRISMA flow diagram

After 4842 unique records were screened, 50 studies met inclusion criteria (figure 1; appendix pp 17–28) for the systematic review. Of these 50 studies, 33 were retrospective cohort, 16 were prospective cohort, and one was a randomised controlled trial (appendix pp 17–28). These studies included 10 460 participants (mean 209, range 45–2248). Based on 47 studies with age information, the median age was 35 years (IQR 33–38). Studies were conducted in 12 countries—Austria,^34^ Australia,^35^, ^36^, ^37^ Belgium,^38^, ^39^, ^40^, ^41^, ^42^ Canada,^43^, ^44^, ^45^, ^46^, ^47^, ^48^, ^49^, ^50^, ^51^ Denmark,^52^, ^53^, ^54^ Finland,^50^, ^55^ Germany,^50^, ^56^ Japan,^57^ Netherlands,^3^, ^58^, ^59^, ^60^, ^61^, ^62^, ^63^, ^64^, ^65^, ^66^, ^67^ Sweden,^50^, ^68^, ^69^, ^70^, ^71^, ^72^, ^73^, ^74^ the UK,^75^, ^76^, ^77^, ^78^, ^79^, ^80^ and the USA^81^, ^82^—all high-income economies (appendix p 4).^83^

The outcome of post-discharge interpersonal violence and crime was most often measured by new criminal offences or recidivism, as reported in 47 (94%) of the 50 studies and 9966 (95%) participants. Typically, recidivism was defined as new convictions (28 [56%] of 50) or criminal charges (five [10%] of 50), as recorded in criminal or police databases. One study defined recidivism on the basis of sentencing data, and six studies did not report how recidivism was defined. Three studies measured post-discharge interpersonal violence and crime using informant or self-report data on physical aggression or violent behaviour. For results synthesis, outcomes were categorised into violent recidivism, general or any recidivism, and sexual recidivism, in line with current research. Shared outcome definitions allowed for comparison of the predictive performance across studies. 27 [54%] of 50 studies assessed multiple types of recidivism; in these cases, all types of recidivism were extracted and included in analysis. Sexual offences were most often included in definitions of violent recidivism, so violent recidivism (including sexual offences) was extracted and included in analysis.

36 risk assessment instruments were examined (appendix p 16). Each risk assessment instrument had between one and five studies assessing predictive validity, apart from the PCL-SV with six studies, the Static-99 and Violence Risk Appraisal Guide (VRAG) with eight studies, the PCL-R with 13 studies, and the HCR-20 version 2 with 16 studies. The most common performance statistic reported was the AUC. Nine studies assessed tool sensitivity, specificity, or positive and negative predictive values.^35^, ^40^, ^42^, ^56^, ^68^, ^69^, ^71^, ^74^, ^80^ One study reported calibration.^74^ In 46 studies, follow-up periods were reported, which ranged from 3 months to 16 years; nine (20%) of 46 studies reported predictive performance at different timepoints. To produce a weighted average of the predictive performance of risk assessment instruments, a meta-analysis was performed.

**Figure 2:**
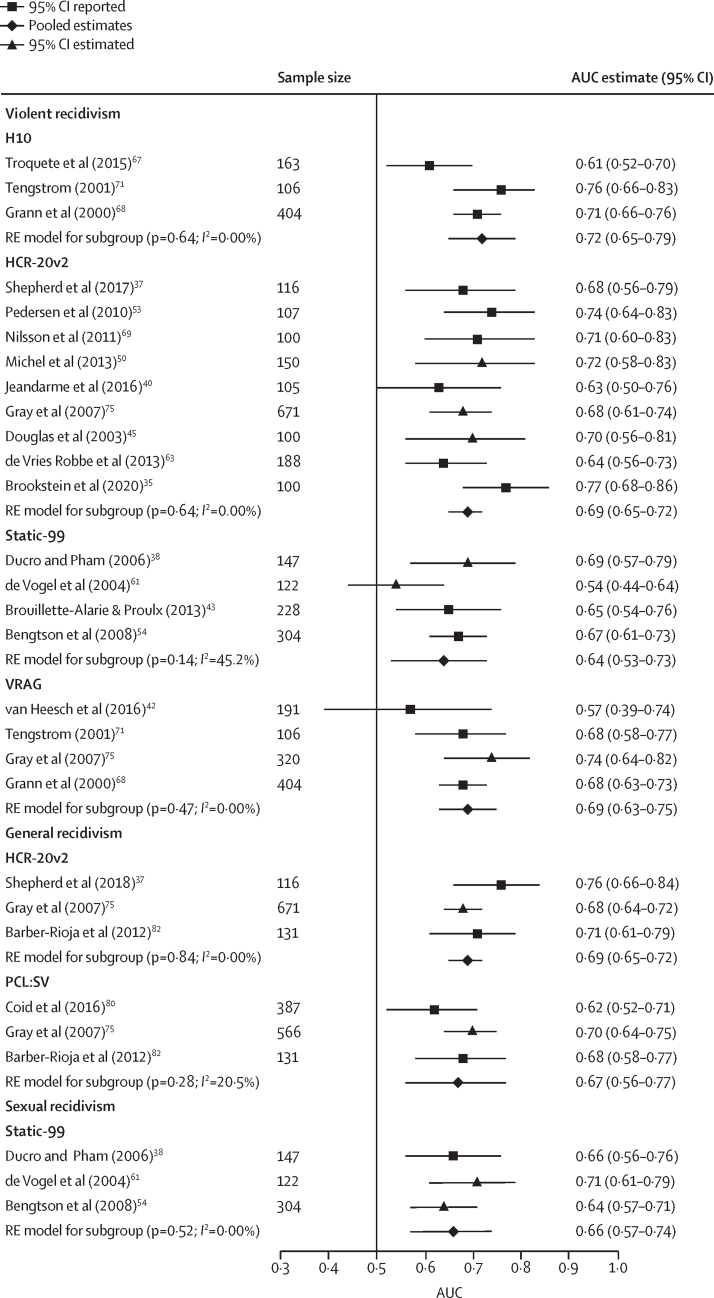
Meta-analysis of independent validation studies with a sample size of more than 100 participants Meta-analyses by outcome type (violent, general, and sexual recidivism) and by risk assessment instrument. AUC=area under curve. HCRv2=Historial, Clinical, Risk Management-20 version 2. PCL:SV=Psychopathy Checklist—Screening Version. RE=random effects. VRAG=Violence Risk Appraisal Guide.

For the primary outcome of violent recidivism, we meta-analysed 20 independent external validations with more than 100 participants drawn from 17 separate studies (figure 2). These validations assessed four tools for violent recidivism (H10, HCR-20 version 2, Static-99, and VRAG). The HCR-20 version 3 did not meet criteria for meta-analysis. The HCR-20 version 2 was assessed in nine studies with AUC ranging from 0·63 to 0·77, and a pooled estimate of 0·69 (95% CI 0·65—0·72; *I*^2^=0%). Four studies investigated the VRAG, with AUC ranging from 0·57 to 0·74, and a pooled estimate of 0·69 (95% CI 0·63—0·75; *I*^2^=0%). Four studies examined the Static-99, with AUC ranging from 0·54 to 0·69, and a pooled estimate of 0·64 (95% CI 0·53–0·73; *I*^2^=45%). Three studies assessed the H10, a subscale of the HCR-20, with AUC ranging from 0·61 to 0·76, and a pooled estimate of 0·72 (95% CI 0·65—0·79; *I*^2^=0%). For general recidivism (any criminal behaviour), six validations (four studies) testing two tools (HCR-20 version 2 and PCL-SV) had sufficient independent external validation studies to be meta-analysed, with a pooled AUC of 0·69 (HCR-20 version 2; 95% CI 0·65–0·72; *I*^2^=0%) and 0·67 (PCL-SV; 95% CI 0·56–0·77; *I*^2^=21%). Only one tool (Static-99) that examined sexual recidivism was meta-analysed (three studies), with a pooled AUC of 0·66 (95% CI 0·57–0·74; *I*^2^=0%).

In our secondary analysis, we examined all 50 eligible studies, irrespective of authorship, validation, or sample size. In 46 studies looking at violent recidivism, the AUCs ranged from 0·39 to 0·86. The risk assessment tools most commonly investigated were the PCL-R (12 studies), HCR-20 version 2 (12 studies), and VRAG (eight studies; appendix pp 5–6). 20 studies examined general recidivism and 11 reports examined sexual recidivism (appendix pp 7–8). For the nine studies that reported paired measures of classification for violent recidivism, sensitivity values ranged from 0·33 to 0·80, specificity from 0·55 to 0·85, positive predictive value (PPV) from 0·30 to 0·74, and negative predictive value (NPV) from 0·70 to 0·96. For paired measures of classification for general recidivism, sensitivity values ranged from 0·60 to 0·89, specificity from 0·52 to 0·72, PPV from 0·00 to 0·28, and NPV from 0·71 to 0·97. When investigating performance by prediction horizons in a subgroup analysis, there was no difference in AUCs (appendix p 10).

**Figure 3:**
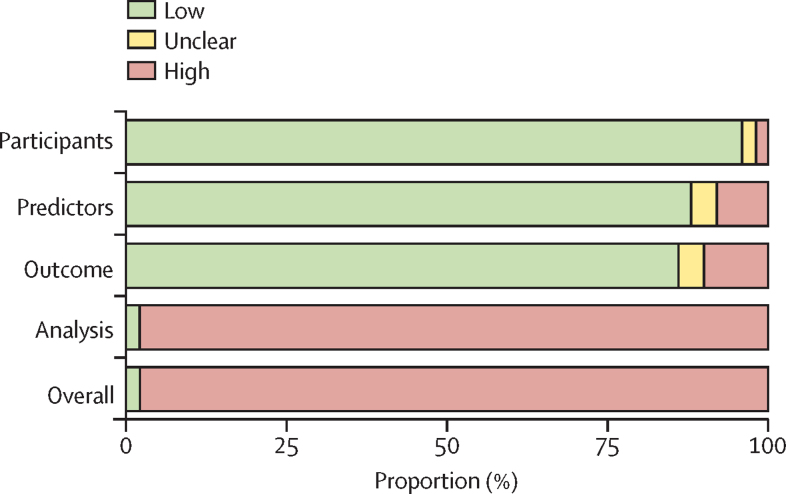
Risk of bias among risk assessment instruments Unweighted bar plot of the distribution of risk-of-bias judgements across all studies within each bias domain.

The risk of bias was high for almost all studies (49 [98%] of 50; figure 3). For individual PROBAST domain ratings, see the appendix (pp 29–30). High rates of bias risk were primarily due to the high risk of bias in the PROBAST analysis domain (49 [98%] of 50) and failure to evaluate the performance appropriately by not assessing instrument calibration (appendix pp 29–30). Only one study of FoVOx^74^ reported calibration metrics. Most studies (47 [94%] of 50) externally validated at least one existing risk assessment instrument in a wholly independent sample (appendix p 9).

In 47 studies, participant sex was specified or could be inferred from ward type. Of these studies, 8710 (88%) of the 9905 participants recruited were men, and 22 (47%) of the 47 studies only recruited male participants. Two studies recruited women only,^59^, ^66^ and one recruited an even ratio of men to women.^51^ The two female-only studies had sample sizes of less than 100 (n=71 and n=45) so were not included in the primary analyses. The female-only samples typically reported lower AUCs than the mostly male samples when assessing the predictive performance of the HCR-20, PCL-R, and Short-Term Assessment of Risk and Treatability instruments (appendix pp 5–7). Of those studies with a mixed sample, only two studies^44^, ^51^ disaggregated predictive validity by sex, with no clear evidence that tools were more predictive when stratified by sex.

## Discussion

This systematic review and meta-analysis examined the predictive performance of risk assessment instruments for violent and criminal outcomes among individuals discharged from forensic psychiatric hospitals. 50 studies were included, involving 10 460 participants from 12 countries. Overall, findings were mixed, with wide variation in study quality, outcome reporting, and the predictive performance of the tools studied. Almost all studies (49 [98%] of 50) were assessed as high risk of bias. Numerous implications follow for the clinical use of these tools, and future research examining the predictive performance of these tools should apply methods that address the methodological limitations of previous studies.

First, the number of performance measures reported by included studies was small; most studies (41 [82%] of 50) reported only the AUC. This discrimination metric gives the probability that for a hypothetical pair of individuals who do and do not develop the outcome of interest (ie, violence or crime perpetration), the tool will assign a higher risk estimate to the individual with the outcome. Importantly, a tool can perform well in measures of discrimination even if it is systematically off-target in its predictions, for example by overestimating risk for everyone. This calibration is a crucial consideration if these absolute risk estimations are used to make clinical decisions. Studies therefore also need to report calibration (the agreement between expected and observed probabilities), which was reported in only one study.^74^

The reporting of paired measures of classification, such as sensitivity and specificity, was also low, presented in nine (18%) of 50 studies. Tool sensitivity and specificity are important when assessing the use of a tool to make specific clinical decisions. The preferred weighting of sensitivity (ie, minimising false negatives) and specificity (ie, minimising false positives) depends on the severity of the outcome being predicted and the nature of the intervention being linked to the assessment. In forensic mental health settings, tools with high sensitivity will be the most relevant from a public safety perspective and could garner more political support, whereas tools with high specificity might best protect the rights of patients.^84^ More research is required to determine which risk assessment instruments exhibit optimal levels of sensitivity and specificity.

Another common methodological problem was small sample sizes, with 19 (38%) of 50 studies recruiting fewer than 100 participants (appendix pp 17–28). As recidivism outcomes are not common in these samples,^85^ small samples lead to uncertain risk estimates. However, obtaining large samples of forensic psychiatric patients is not feasible in many settings. Using multisite or nationwide registry data could address this limitation. Another finding of the current review is that most tools were independently externally validated (although not six newer tools). Independent validations should be prioritised because authorship bias can influence reporting of findings.^86^

The results from the meta-analysis provide evidence on the predictive performance of the most common risk assessment instruments. We found that the H10, HCR-20 version 2, VRAG, and Static-99 perform similarly at predicting violent recidivism, with overlapping CIs and pooled AUCs ranging from 0·64 to 0·72. A secondary analysis of all 50 eligible studies, irrespective of authorship, validation, or sample size, clarified the heterogeneity in performance between different studies and instruments. Although most studies reported AUCs of approximately 0·70, some studies (especially those with small sample sizes) reported a predictive performance not different to chance. This finding suggests that the appraisal of these risk assessment studies needs to pay particular attention to sample size.

The wide range in prediction horizons (follow-up periods ranged from 3 months to 16 years) between studies could moderate predictive performance. Although we did not find evidence of this in our post-hoc analysis (appendix p 10), future work should define outcome windows more clearly. Furthermore, most current literature does not consistently report or account for the provision and intensity of ongoing community supervision, potential readmission to hospital, or incarceration during follow-up. All these factors could potentially limit opportunities to reoffend and reduce outcome rates. Moreover, in contexts where violence risk assessment instruments are used continuously to assess and manage the likelihood of violent outcomes, it is difficult to distinguish whether false-positive errors (ie, a risk assessment predicted that someone is high risk of violence, when they did not have a violent outcome) occurred or whether the management strategies implemented following a risk assessment effectively decreased risk. Therefore, predictive performance will probably be attenuated in circumstances of active risk management. Treatment and risk management procedures should be reported to allow accurate appraisal of risk prediction potential. Current literature often does not acknowledge how the population and context in which risk assessment instruments are developed and validated contribute to predictive performance and its validity in risk management procedures.

Included studies considered a wide range of instruments to assess the risk of interpersonal violence and crime, and tools were often examined for predicting different outcomes to those they were originally developed to assess. For example, the Static-99 (and its revisions), a tool developed to predict sexual recidivism, has been used to assess violent and general recidivism. The second most frequently studied tool was the PCL-R, which was developed to assess psychopathy. As tool predictive performance is highly dependent on population and setting,^87^ accuracy will be poorer if instruments are not used as intended.^88^ The overlapping content between some risk assessment instruments might be relevant to comparing their performance. In addition, some items might be redundant, as they were not tested in multivariate models.

We found that the H10—a HCR-20 subscale that includes 10 historical factors—performed similarly to the HCR-20 full scale. Risk assessments are resource intensive,^22^ so if short-form or tool subscales are equally predictive, using these in clinical services could constitute a more efficient use of resources.

Included studies were typically of male-dominated samples (88% of included participants), and 47% of studies recruited exclusively male participants. The generalisability of tools to female patients could be hampered by differences in the baseline rates of violence between men and women, and in risk factor association with violence. Domains of risk relevant for female populations might include intimate partner violence,^18^ sex work,^89^ pregnancy at a young age,^90^ and self-harm.^91^ Interestingly, the FAM—designed to supplement the HCR-20 version 2 for female forensic patients—did not yield higher AUC values in a female-only sample compared with the HCR-20 version 3.^59^

Poor predictive performance of an assessment tool, or lack of validation, has direct clinical and ethical implications for patients. Discharge from secure settings could be expedited or delayed depending on the findings of a risk assessment instrument. Inaccurate risk prediction resulting in extended detention can be harmful. Furthermore, inaccurate prediction resulting in the discharge of a patient who subsequently reoffends will disrupt the clinical care of the patient and harm public health and safety. Therefore, to avoid these negative outcomes, ensuring that the risk assessment tools implemented in clinical practice have high predictive performance and perform consistently in varied contexts is important for ethical conduct. Some research studies have labelled AUC values in categories, but this is not recommended.^92^ Instruments can be compared on discriminative ability using AUC values, with the best AUC implying greater discrimination performance; however, additional measures are needed to verify the potential clinical positive effect. In the case of this review, the risk assessment instrument with the best predictive performance was the H10 (pooled AUC of 0·72). One overall implication is that risk assessment instruments do discriminate better than chance, and other research suggests that tools are typically more accurate than unstructured clinical decision making, especially in predicting violence.^93^ What constitutes adequate performance is dependent on the context of application;^94^ therefore, the AUC values of risk assessment instruments in this field cannot, in a straightforward manner, be compared with other domains (eg, in cancer or cardiovascular medicine), since the predictors and outcomes are different.

For forensic mental health services, the findings suggest that, as a minimum, risk assessment tools should be used to complement clinical decision making; they should not be used to inform decisions about length of stay without validations showing high sensitivity. With evidence of high specificity, risk assessment tools can be included in discussions about discharge planning, particularly about how to allocate follow-up resources. New tools should not be introduced to services without information on these classification measures and calibration. In addition, any tools developed without multivariable models or inclusion of the strongest risk factors (ie, age, sex, and previous antisocial or violent behaviour) are very unlikely to be accurate in new settings. Other considerations include the tool being developed on forensic mental health populations, evidence of feasibility and acceptability, the individual variables making up the tool being weighted, and internal validation.^95^ Furthermore, instruments that facilitate formulation as part of the risk assessment procedure might aid clinicians in creating effective risk management plans that are sensitive to risk erosion, actively mitigate risk, and avert violence.

To our knowledge, this is the first comprehensive systematic review and meta-analysis of the performance of risk assessment instruments for post-discharge interpersonal violence and crime in forensic mental health specifically. One of the review's limitations is that the risk of bias (PROBAST) tool was developed for prediction models in general medicine, rather than risk assessment instruments in forensic mental health, and the threshold for bias might be too low for studies reporting on crime outcomes. This limitation has resulted in high risk of bias across studies, with little granularity on how bias differs between studies; and a sensitivity analysis was not possible to examine whether risk of bias was associated with the predictive validity of the tools.

Taken together, the findings provide some directions for future work. From a methodological perspective, future research should be adequately powered and report multiple estimates of predictive performance to inform clinical decisions. These metrics should include calibration, sensitivity, specificity, and positive and negative predictive values. All available research was from high-income countries. Future research should seek to include women and samples from low-income and middle-income countries, and investigate the incremental value of novel risk factors. Another research direction is to examine to what extent performance can be improved by incorporating novel risk factors. For example, markers of previous neurological damage or head trauma could improve performance, as neurobiological stress and injury can cause emotional and behavioural changes, increasing the risk of violence.^96^

In summary, the performance of current tools at predicting the risk of interpersonal violence and crime in forensic mental health is mixed, with performance varying between instruments. Most investigations solely reported the AUC for model performance, and failed to present other key measures, such as calibration, sensitivity, and specificity. Higher quality risk assessment tools could contribute to better risk management in forensic mental health.

## Data sharing

Individual participant data are not available. The study protocol was published with PROSPERO (CRD42022304716) and is available at https://www.crd.york.ac.uk/PROSPERO/display_record.php?RecordID=304716.

## Declaration of interests

SF has published research on risk assessment, including as part of a team that has derived and validated one tool for predicting violent crime on discharge from secure psychiatric hospitals (FoVOx). All other authors have no competing interests.
