# Supplementary material for: Violence risk assessment instruments in forensic psychiatric populations: a systematic review and meta-analysis
Source: Lancet Psychiatry. 2023 Oct;10(10):780–9. doi: 10.1016/S2215-0366(23)00256-0 (PMC10914679; doi:10.1016/S2215-0366(23)00256-0)
Supplement: Supplementary appendix [file mmc1.pdf]

THE LANCET
Psychiatry

Supplementary appendix

This appendix formed part of the original submission and has been peer reviewed. We post it as supplied by the authors.

Supplement to: Ogonah MCT, Seyedsalehi A, WhitingD, Fazel S. Violence risk assessment instruments in forensic psychiatric populations: a systematic review and meta-analysis. *Lancet Psychiatry* 2023; **10:** 780–89.

Supplementary Material

Violence Risk Assessment Instruments in Forensic Psychiatric Populations: A Systematic Review and Meta-Analysis of their Performance

Supplementary Methods

Appendix 1. Inclusion and exclusion criteria... Page 2

Appendix 2. Supplementary methods………………………………………………………………….. Page 3

Supplementary Figures

Figure 1. Geographical coverage of reviewed study samples... Page 4

Figure 2. Area under the curve statistics for all validations for risk assessment tools used to predict violent recidivism……………………………………………………………………………………………….. Page 5—6

Figure 3. Area under the curve statistics for all validations for risk assessment tools used to predict general recidivism…………………………...…………………………………………………………………….. Page 7

Figure 4. Area under the curve statistics for all validations for risk assessment tools used to predict sexual recidivism……………………………………………………………………………………………….. Page 8

Figure 5. Breakdown of risk of bias: PROBAST analysis domain items Page 9

Figure 6. Apparent, internal, and external validation efforts... Page 9

Figure 7. Time-at-risk for all validations for risk assessment tools used to predict violent recidivism…… Page 10

Supplementary Tables

Table S1. PRISMA checklist... Page 11—14

Table S2. Search strategy and terms……………………………………………….................................... Page 15

Table S3. Risk assessment instruments used to predict post-discharge violence ………................................ Page 16

Table S4. Study characteristics table .... Page 17—28

Table S5. Quality assessment (risk of bias) ratings for reviewed studies... Page 29—30

Supplementary References………………………………………………………………………………. Page 31—36

Appendix 1. Inclusion and exclusion criteria

Inclusion criteria:

- Population: the population of interest is adult forensic psychiatric patients or psychiatric patients admitted to secure/intensive units following violent or criminal incidents. Samples including offenders referred for forensic psychiatric evaluation (FPE) were included as part of this review. In certain countries, FPEs are conducted for defendants suspected of a mental disorder or mental retardation for whom extenuating circumstances may be considered. The FPE is accomplished by a psychiatrist and includes a range of psychological tests and examinations. Typically, FPE involves a 4-week admission whilst the defendant is undergoing assessment; circumstances equivalent to that of a forensic ward, hence why FPE samples have been included in this review. It was required that each study analyzed forensic psychiatric patient cohorts independently, rather than combined with other correctional or general psychiatric samples.
- Intervention: studies that assessed the utility and accuracy of risk assessment instruments in predicting subsequent arrests or reconvictions post-discharge were included. Studies that reported the accuracy of instruments at predicting interpersonal violence or crime which resulted in recall back into forensic psychiatric institutions, or crime-related incidents, i.e., incidents coded under offending categories, were included regardless of if patients were prosecuted or sentenced.
- Material and design: both retrospective and prospective studies were included.
- Analysis: studies that met pre-determined statistical standards for assessing predictive validity were included. Appropriate and accepted statistical techniques included calibration, specificity, sensitivity, positive prediction values (PPVs), negative prediction values (NPVs), c-index and/or area under the (receiver operating) curve (AUC) for the risk instrument total score. When both the low-medium and medium-high cut-off's have been presented, the sensitivity, specificity, PPV, and NPV for the medium-high cut-off have been chosen as the discrimination metric. When both the total score and final risk judgement/summary risk rating/risk category have been presented, total scores were chosen as the measure of predictive validity as they are the most consistently reported measure.

  The AUC is widely used as a global discrimination metric[1] which expresses the probability that a randomly selected recidivist scores higher on a risk assessment instrument, compared to a non-recidivist. In terms of discrimination, an AUC equals 1 for a perfect test and 0.5 for a completely uninformative test (i.e., discrimination no better than chance). When studies have reported two different AUC values, one calculated using information available on admission and one using information available on discharge, the value calculated using information available at discharge has been selected. When studies have reported both violent and non-sexual violent outcomes, the more inclusive outcome (violent recidivism), has been selected for inclusion and was extracted. Further, as many included studies failed to report the 95% confidence interval of their AUC, missing confidence intervals were estimated based on the number of recidivists and non-recidivists in the sample.[2,3]

Exclusion criteria:

- Population: studies that focused on general correctional samples or general psychiatric patients were excluded from the review.
- Material and design: systematic reviews and book reviews were excluded from the review. Case-control studies[4] have been excluded as they cannot be used to estimate absolute risks, leading to incorrect estimates of baseline hazard. Prediction model (or machine learning model) studies have been excluded from this review unless they have developed a risk assessment instrument based on their prediction model.
- Analysis: studies which only assess the predictive validity of change scores (posttreatment scores minus pretreatment scores;[5] have been excluded from the review.

Appendix 2. Supplementary Methods

Data-analysis

For those studies which reported multiple follow-up periods, the longest follow-up period was extracted for data analysis – as there was not a common level of follow-up. When a tool had been validated at least three times for the outcome, we applied a random-effects model, using the inverse-variance method, for pooling the logit transformation of the area under the ROC curve and confidence intervals. AUC values were meta-analysed, as they were the only commonly reported metric. A random effects model was chosen as we anticipated considerable between-study heterogeneity, in part due to variable prediction horizons. The discrimination metric (AUC) was estimated on the logit scale, to ensure between-study normality,[6] and then backtransformed for ease of interpretation. The restricted maximum likelihood estimator[7] was used to calculate the heterogeneity variance τ^2. We used Knapp-Hartung adjustments[8] to calculate the confidence interval around the pooled effect. Analyses were performed with R version 4.1.0[9] using the metafor[10] and meta-package.[11, 12] The dmetar-package was used to detect outliers and influential cases.[13] The Risk of Bias figure was created using the robvis-package.[14] The world map figure was created using the maps and ggplot2 packages in R.

We note that for the meta-analysis, the number of validations may not be the same as the number of studies because (a) a single study could have validations of different tools, which were meta-analysed separately, (b) some studies with external validations of n > 100 may not be included in the meta-analysis, as there needed to be three external validations with n > 100 for each tool to be included.

We decided to pool all risk assessment instruments regardless of study design as while the performance of a risk assessment instrument can be affected by differences in design,[15] this is one of the many study-level characteristics that contribute to variation between the results of validation studies (with other sources of heterogeneity including patient case-mix, outcome definition, and follow-up duration), and we have accounted for these by using a random (rather than fixed) effects meta-analysis model.[16] Further, we have chosen to include all study designs, and not weigh one study design more than others. According to the CHARMS checklist[17] data from both retrospective and prospective cohorts, nested case-control, and case-cohort studies can be used for prediction model development and validation, and RCTs, do not provide a higher quality of evidence for this purpose as their restrictive criteria for entry can hamper the generalisability of prediction models.

The Prediction model Risk of Bias Assessment Tool (PROBAST), was adapted and provided a risk of bias (RoB) rating for each study, with low, high, or unclear risk of bias categorisations. Item 3·3 ('Were predictors excluded from the outcome definition?') was deemed not applicable to the scope of this review, therefore, was excluded when reaching an overall judgement about the risk of bias of the instrument. A domain where all signalling questions are answered as "yes" or "probably yes" is judged as "low RoB". Any answers of "no" or "probably no" for one or more questions results in a high risk of bias in that domain.

Figure 1. Geographical coverage of reviewed study samples

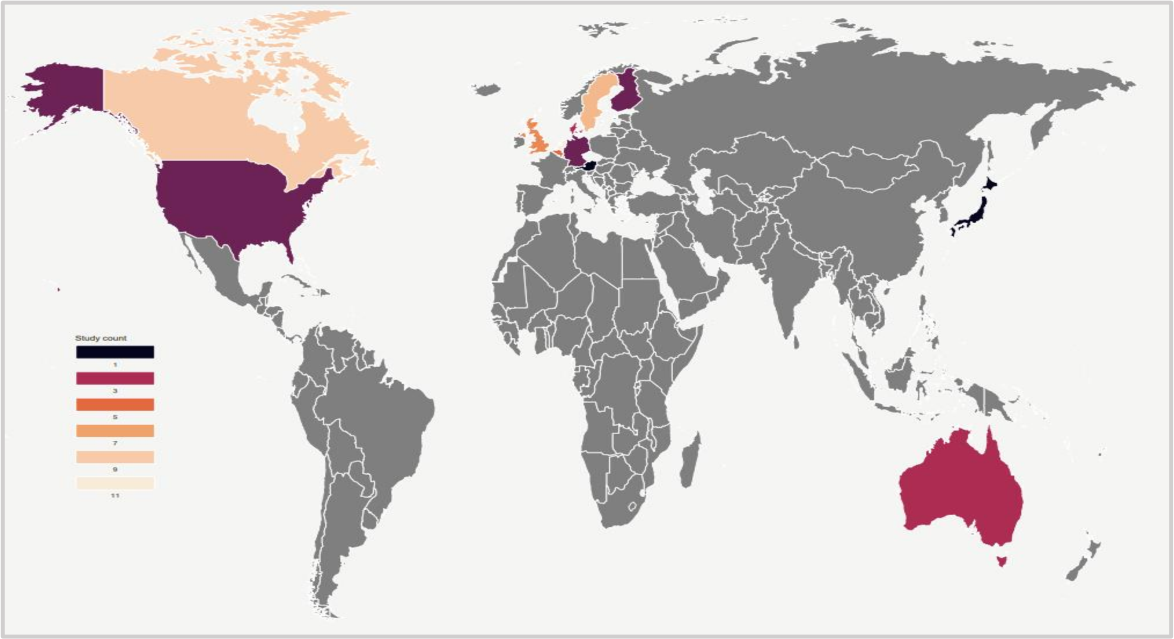

Figure 2. Area under the curve statistics for all validations for risk assessment tools used to predict violent recidivism (including non-independent and studies with small sample sizes)

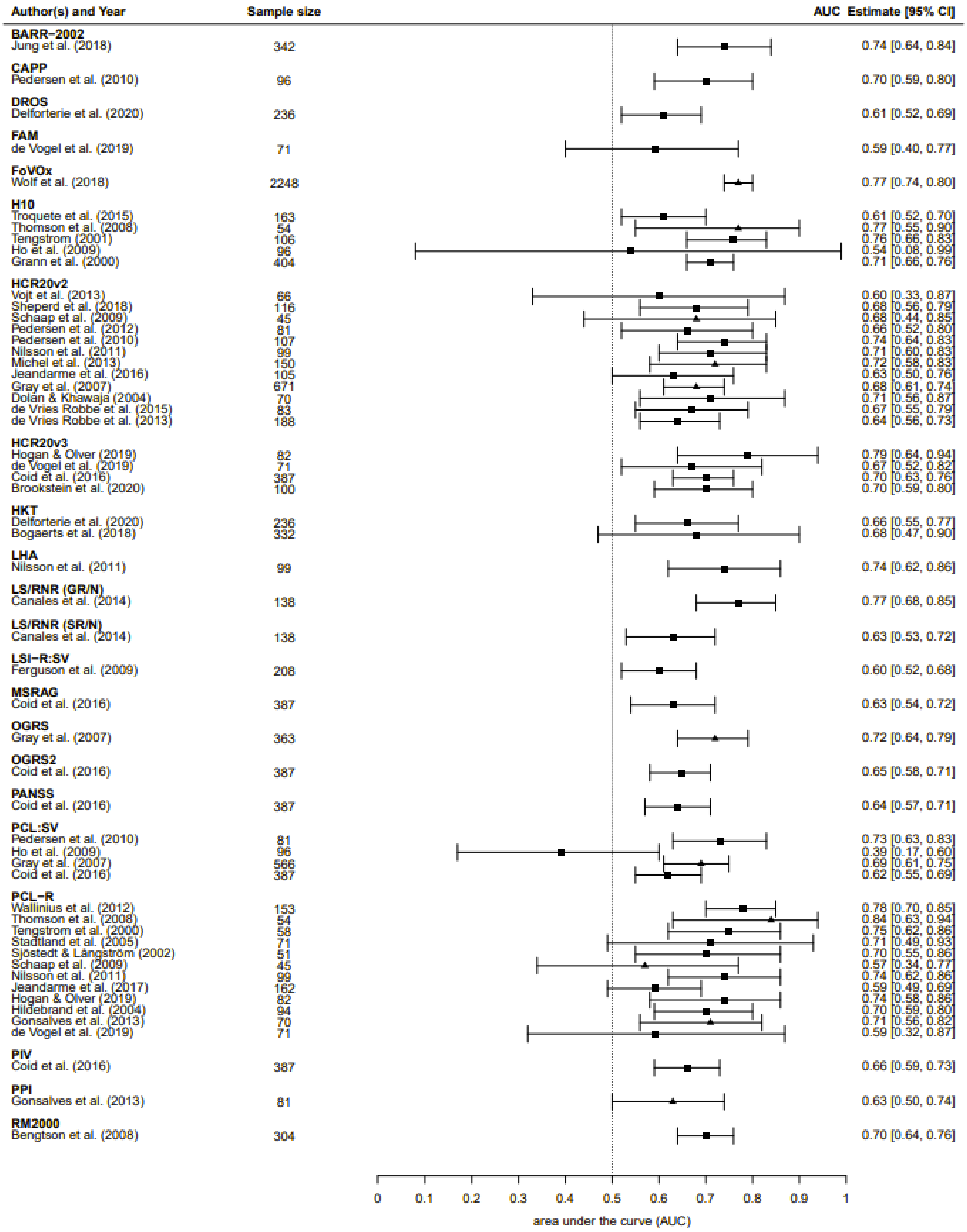

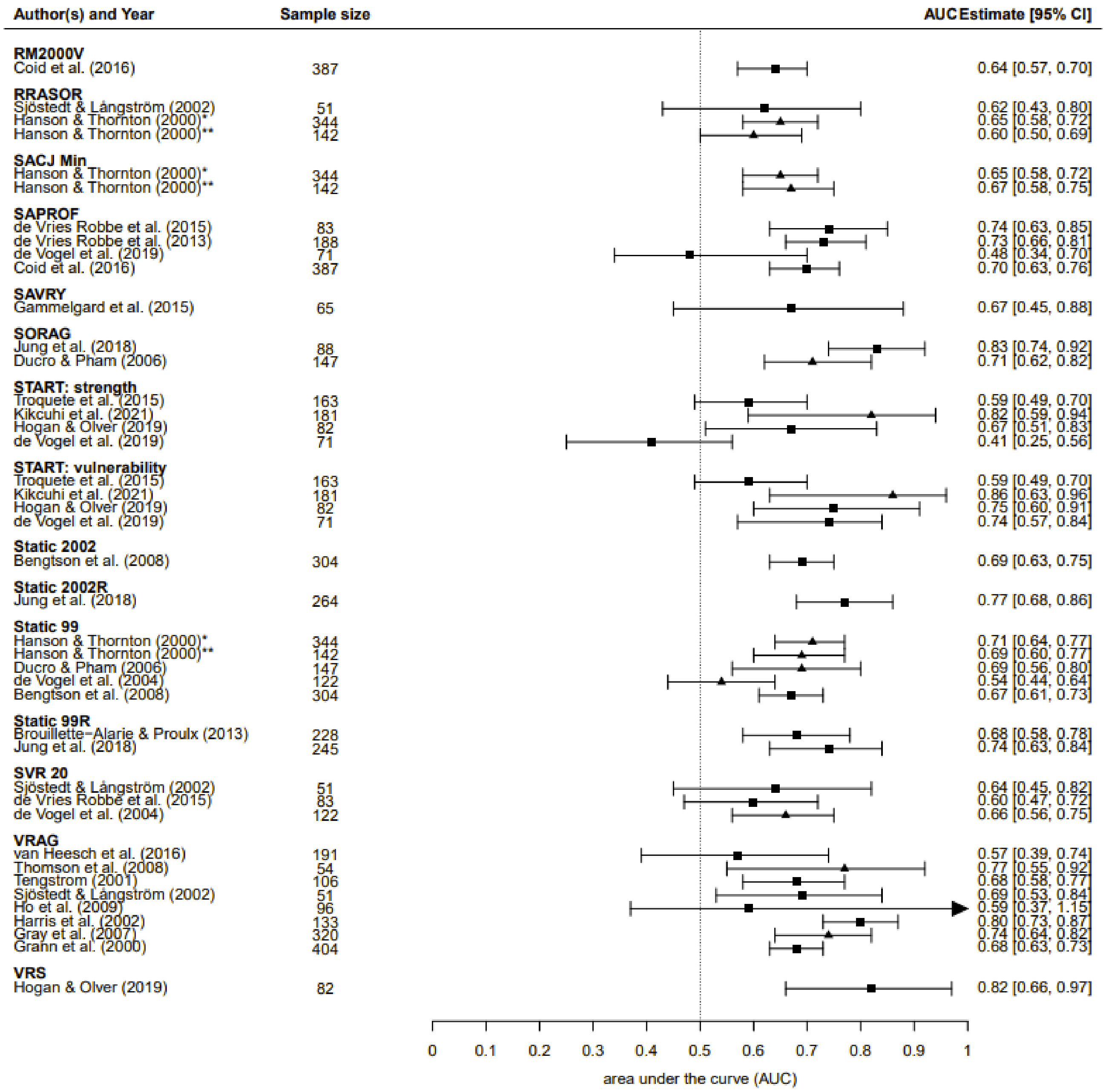

Note. Two studies[18,19] did not report the observed recidivism level or the AUC confidence intervals, therefore, could not be represented on the forest plot.

*Hanson & Thornton (2000) includes the PPI sample; **Hanson & Thornton (2000) includes the Oak Ridge sample. The predictive performance of the risk assessment tools was reported separately for each sample.

If a study assessed the predictive performance of multiple versions of the same tool, only the most up-to-date version is represented.

■ = 95% CI reported; ▲ = 95% CI estimated

Figure 3. Area under the curve statistics for all validations for risk assessment tools used to predict general recidivism

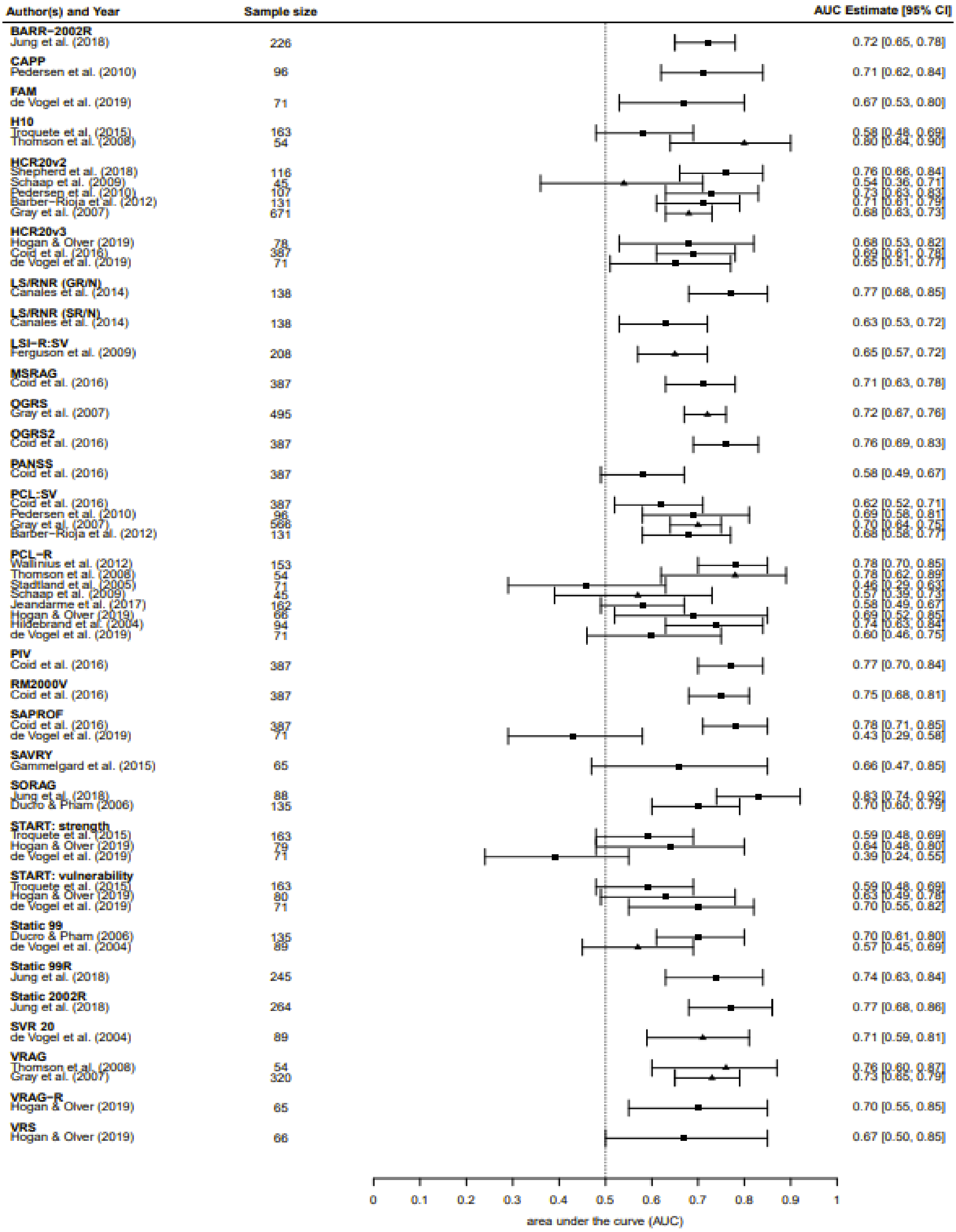

Note. Two studies [19,20] did not report the observed recidivism level or the AUC confidence intervals, therefore, could not be represented on the forest plot.

If a study assessed the predictive performance of multiple versions of the same tool, only the most up-to-date version of each tool is represented.

■ = 95% CI reported; ▲ = 95% CI estimated

Figure 4. Area under the curve statistics for all validations for risk assessment tools used to predict sexual recidivism

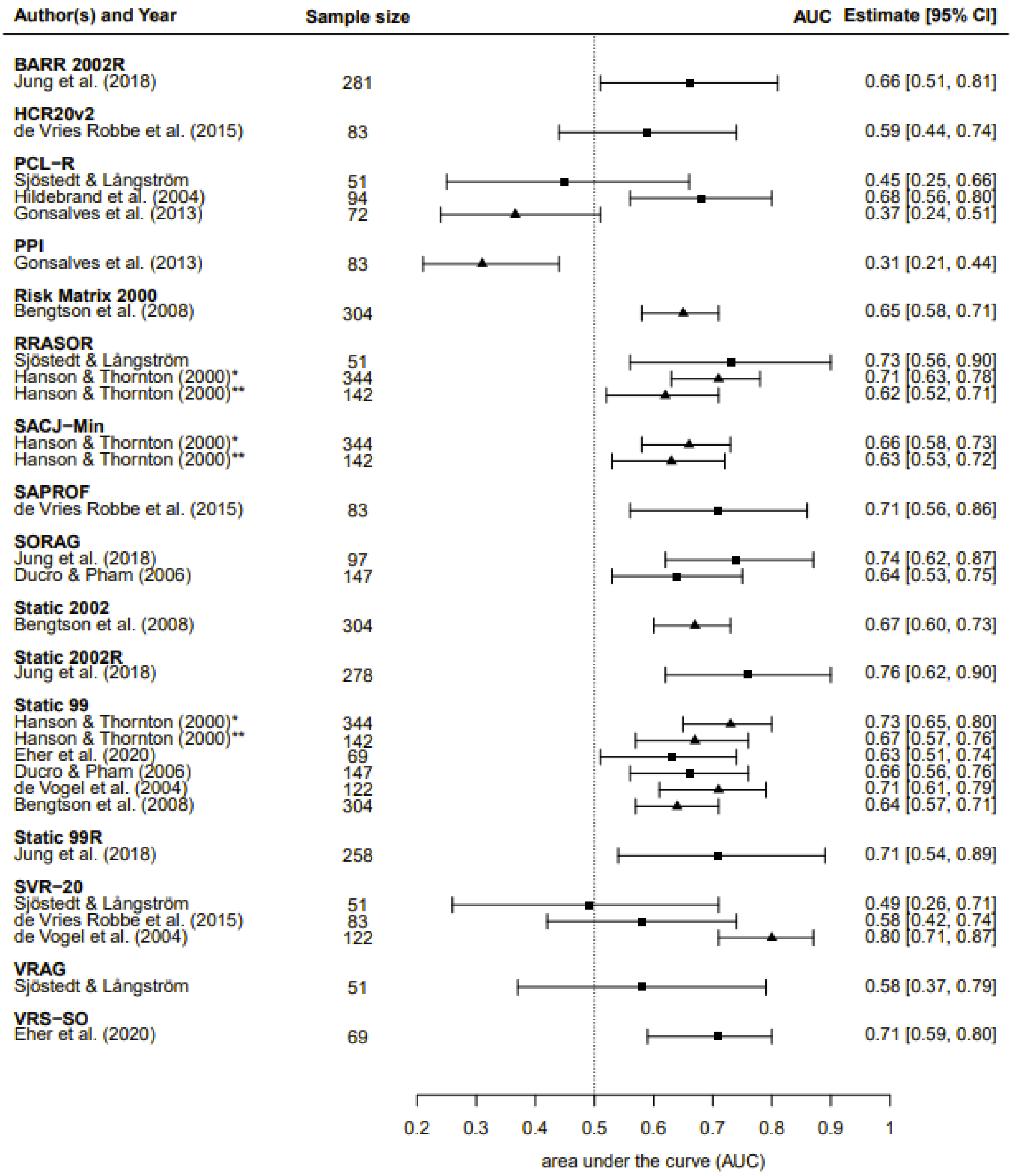

Note. One study[19] did not report the observed recidivism level or the AUC confidence intervals, therefore, could not be represented on the forest plot.

*Hanson & Thornton (2000) includes the PPI sample; **Hanson & Thornton (2000) includes the Oak Ridge sample. The predictive performance of the risk assessment tools was reported separately for each sample.

If a study assessed the predictive performance of multiple versions of the same tool, only the most up-to-date version of each tool is represented.

■ = 95% CI reported; ▲ = 95% CI estimated

Figure 5. Breakdown of risk of bias: PROBAST analysis domain items

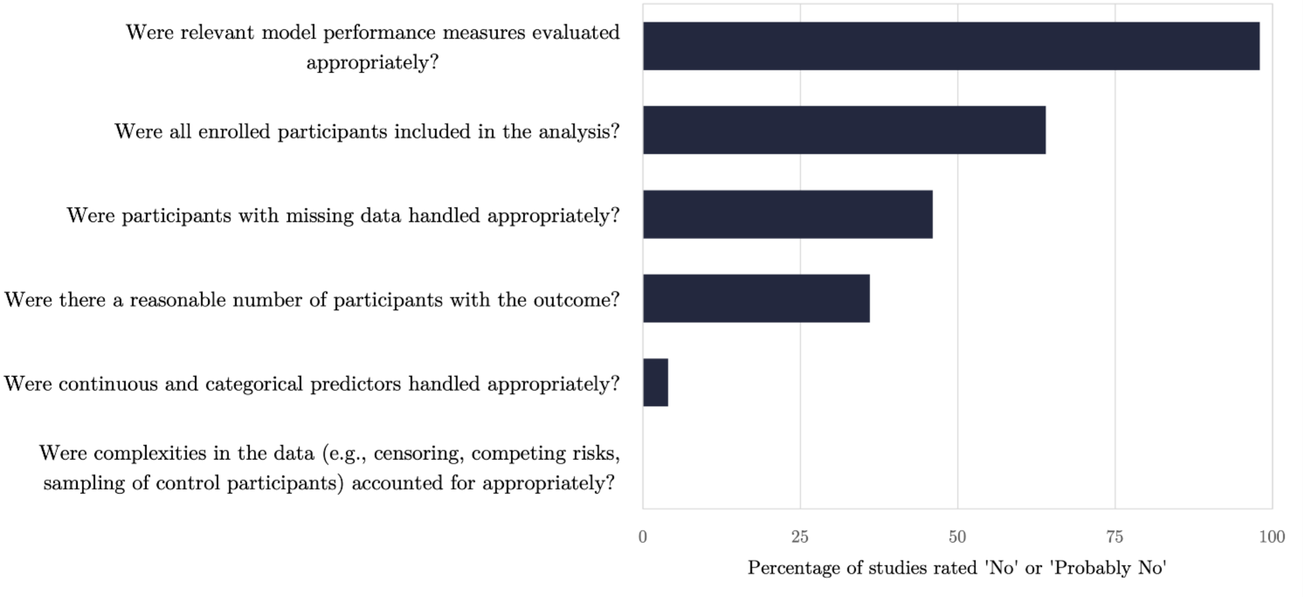

Figure 6. Apparent, internal, and external validation efforts

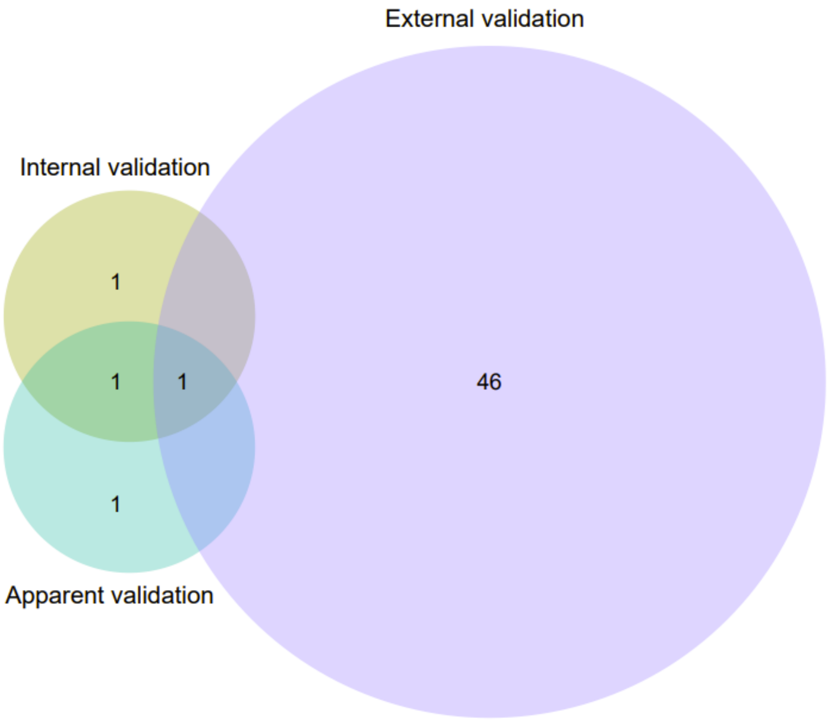

Figure 7. Time-at-risk for all validations of risk assessment tools used to predict violent recidivism

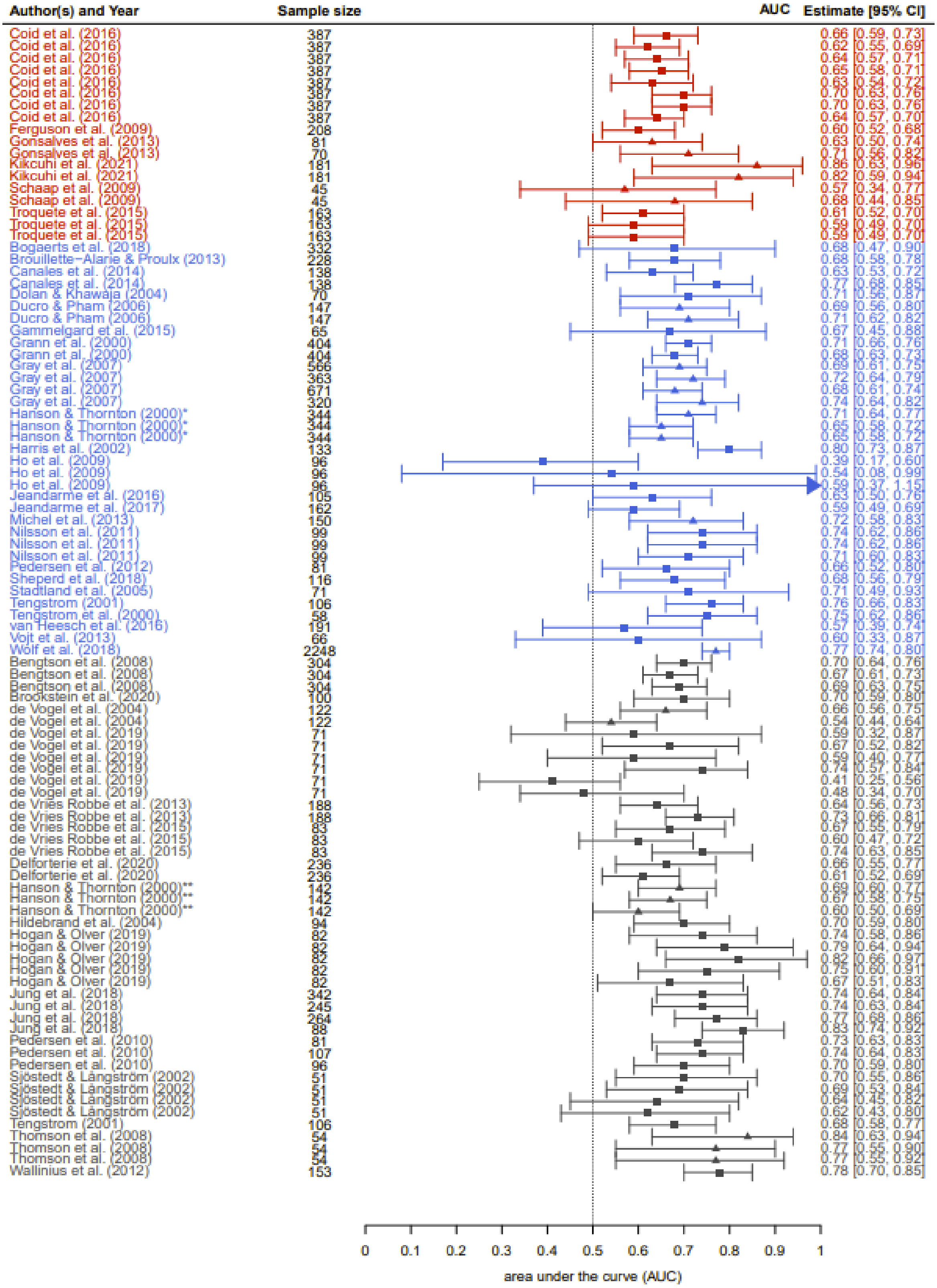

Area under the curve statistics for all studies for risk assessment tools used to predict violent recidivism (including non-independent and studies with small sample sizes). Here we represent the different time-at-risk for each individual study. Studies with a follow-up duration of 12 months or less are coloured in red, studies with a follow-up time of between 1-5 years are coloured in blue, and studies with a follow-up duration of over 5 years are represented in grey.

Table S1. PRISMA checklist

PRISMA 2020 Main Checklist

Topic	No.	Item	Location where item is reported
**TITLE**			
**Title**	1	Identify the report as a systematic review.	Title page
**ABSTRACT**			
**Abstract**	2	See the PRISMA 2020 for Abstracts checklist	
**INTRODUCTION**			
**Rationale**	3	Describe the rationale for the review in the context of existing knowledge.	Introduction, para 2 & 3
**Objectives**	4	Provide an explicit statement of the objective(s) or question(s) the review addresses.	Introduction, para 5
**METHODS**			
**Eligibility criteria**	5	Specify the inclusion and exclusion criteria for the review and how studies were grouped for the syntheses.	Methods, search strategy and selection criteria, para 3
**Information sources**	6	Specify all databases, registers, websites, organisations, reference lists and other sources searched or consulted to identify studies. Specify the date when each source was last searched or consulted.	Methods, search strategy and selection criteria, para 2
**Search strategy**	7	Present the full search strategies for all databases, registers and websites, including any filters and limits used.	Supplementary Table S2
**Selection process**	8	Specify the methods used to decide whether a study met the inclusion criteria of the review, including how many reviewers screened each record and each report retrieved, whether they worked independently, and if applicable, details of automation tools used in the process.	Methods, search strategy and selection criteria, para 4
**Data collection process**	9	Specify the methods used to collect data from reports, including how many reviewers collected data from each report, whether they worked independently, any processes for obtaining or confirming data from study investigators, and if applicable, details of automation tools used in the process.	Methods, data analysis, para 1
**Data items**	10a	List and define all outcomes for which data were sought. Specify whether all results that were compatible with each outcome domain in each study were sought (e.g. for all measures, time points, analyses), and if not, the methods used to decide which results to collect.	Supplementary Appendix 1
	10b	List and define all other variables for which data were sought (e.g. participant and intervention characteristics, funding sources). Describe any assumptions made about any missing or unclear information.	Study Characteristics Table S3

Topic	No.	Item	Location where item is reported
**Study risk of bias assessment**	11	Specify the methods used to assess risk of bias in the included studies, including details of the tool(s) used, how many reviewers assessed each study and whether they worked independently, and if applicable, details of automation tools used in the process.	Methods, data analysis, para 4
**Effect measures**	12	Specify for each outcome the effect measure(s) (e.g. risk ratio, mean difference) used in the synthesis or presentation of results.	Methods, data analysis, para 2
**Synthesis methods**	13a	Describe the processes used to decide which studies were eligible for each synthesis (e.g. tabulating the study intervention characteristics and comparing against the planned groups for each synthesis (item 5)).	Methods, data analysis, para 2 & 3
	13b	Describe any methods required to prepare the data for presentation or synthesis, such as handling of missing summary statistics, or data conversions.	Methods, data analysis, para 2; Supplementary Appendix 2
	13c	Describe any methods used to tabulate or visually display results of individual studies and syntheses.	Supplementary Appendix 2
	13d	Describe any methods used to synthesize results and provide a rationale for the choice(s). If meta-analysis was performed, describe the model(s), method(s) to identify the presence and extent of statistical heterogeneity, and software package(s) used.	Methods, data analysis para 2 & 3; Supplementary Appendix 2
	13e	Describe any methods used to explore possible causes of heterogeneity among study results (e.g. subgroup analysis, meta-regression).	na
	13f	Describe any sensitivity analyses conducted to assess robustness of the synthesized results.	na
**Reporting bias assessment**	14	Describe any methods used to assess risk of bias due to missing results in a synthesis (arising from reporting biases).	na
**Certainty assessment**	15	Describe any methods used to assess certainty (or confidence) in the body of evidence for an outcome.	na
**RESULTS**			
**Study selection**	16a	Describe the results of the search and selection process, from the number of records identified in the search to the number of studies included in the review, ideally using a flow diagram.	Figure 1
	16b	Cite studies that might appear to meet the inclusion criteria, but which were excluded, and explain why they were excluded.	Supplementary Appendix 1
**Study characteristics**	17	Cite each included study and present its characteristics.	Study Characteristics Table S4
**Risk of bias in studies**	18	Present assessments of risk of bias for each included study.	Supplementary Table S5
**Results of individual studies**	19	For all outcomes, present, for each study: (a) summary statistics for each group (where appropriate) and (b) an effect estimate and its precision (e.g. confidence/credible interval), ideally using structured tables or plots.	Supplementary Figures 2-4
**Results of syntheses**	20a	For each synthesis, briefly summarise the characteristics and risk of bias among contributing studies.	Results, para 1 & 6
	20b	Present results of all statistical syntheses conducted. If meta-analysis was done, present for each the summary estimate and its precision (e.g. confidence/credible interval) and measures of statistical heterogeneity. If comparing groups, describe the direction of the effect.	Results, para 4

Topic	No.	Item	Location where item is reported
	20c	Present results of all investigations of possible causes of heterogeneity among study results.	Figure 3
	20d	Present results of all sensitivity analyses conducted to assess the robustness of the synthesized results.	na
**Reporting biases**	21	Present assessments of risk of bias due to missing results (arising from reporting biases) for each synthesis assessed.	na
**Certainty of evidence**	22	Present assessments of certainty (or confidence) in the body of evidence for each outcome assessed.	na
**DISCUSSION**			
**Discussion**	23a	Provide a general interpretation of the results in the context of other evidence.	Discussion. para 1
	23b	Discuss any limitations of the evidence included in the review.	Discussion, para 2-4
	23c	Discuss any limitations of the review processes used.	Discussion, para 12
	23d	Discuss implications of the results for practice, policy, and future research.	Discussion, para 11
**OTHER INFORMATION**			
**Registration and protocol**	24a	Provide registration information for the review, including register name and registration number, or state that the review was not registered.	Methods, search strategy and selection criteria, para 4
	24b	Indicate where the review protocol can be accessed, or state that a protocol was not prepared.	Methods, search strategy and selection criteria, para 4
	24c	Describe and explain any amendments to information provided at registration or in the protocol.	Methods, changes from the protocol
**Support**	25	Describe sources of financial or non-financial support for the review, and the role of the funders or sponsors in the review.	Methods, role of the fundung source, and acknowledgements
**Competing interests**	26	Declare any competing interests of review authors.	Declarations of interest
**Availability of data, code and other materials**	27	Report which of the following are publicly available and where they can be found: template data collection forms; data extracted from included studies; data used for all analyses; analytic code; any other materials used in the review.	Data sharing

PRIMSA Abstract Checklist

Topic	No.	Item	Reported?
**TITLE**			
**Title**	1	Identify the report as a systematic review.	Yes
**BACKGROUND**			
**Objectives**	2	Provide an explicit statement of the main objective(s) or question(s) the review addresses.	Yes
**METHODS**			
**Eligibility criteria**	3	Specify the inclusion and exclusion criteria for the review.	Yes
**Information sources**	4	Specify the information sources (e.g. databases, registers) used to identify studies and the date when each was last searched.	Yes
**Risk of bias**	5	Specify the methods used to assess risk of bias in the included studies.	Yes
**Synthesis of results**	6	Specify the methods used to present and synthesize results.	Yes
**RESULTS**			
**Included studies**	7	Give the total number of included studies and participants and summarise relevant characteristics of studies.	Yes
**Synthesis of results**	8	Present results for main outcomes, preferably indicating the number of included studies and participants for each. If meta-analysis was done, report the summary estimate and confidence/credible interval. If comparing groups, indicate the direction of the effect (i.e. which group is favoured).	Yes
**DISCUSSION**			
**Limitations of evidence**	9	Provide a brief summary of the limitations of the evidence included in the review (e.g. study risk of bias, inconsistency and imprecision).	Yes
**Interpretation**	10	Provide a general interpretation of the results and important implications.	Yes
**OTHER**			
**Funding**	11	Specify the primary source of funding for the review.	Yes
**Registration**	12	Provide the register name and registration number.	Yes

From: Page MJ, McKenzie JE, Bossuyt PM, Boutron I, Hoffmann TC, Mulrow CD, et al. The PRISMA 2020 statement: an updated guideline for reporting systematic reviews. MetaArXiv. 2020, September 14. DOI: 10.31222/osf.io/v7gm2. For more information, visit: www.prisma-statement.org

Table S2. Search strategy and terms

DATABASE: PsycINFO, Embase, Medline, Global Health

**Search terms**		
**1**	Subject headings	Risk Assessment
	Keyword	(Risk assess* OR "Short-Term Assessment of Risk and Treatability" OR START OR "Structured Assessment of Protective Factors" OR SAPROF OR "Violence Risk Screening-10" OR V-Risk-10 OR "Historical Clinical Risk Management-20" OR HCR-20 OR "Psychopathy Checklist Revised" OR PCL-R OR "Psychopathy Checklist Screening Version" OR PCL:SV OR "Level of Service Inventory Revised" OR LSI-R OR "Violence Risk Scale" OR VRS OR "Violence Risk Appraisal Guide" OR VRAG OR "Forensisch Operationalisiertes Therapie- und Risiko-Evaluations System" OR FOTRES OR "Historische Klinische, Toekomstige-20" OR HKT-30 OR "Sexual Violence Risk - 20" OR SVR-20 OR "Sex Offender Risk Appraisal Guide" OR SORAG OR Static 99)
		AND
**2**	Subject headings	Predictive accuracy
	Keywords	(Predict* OR accura* OR valid* OR reliab* OR "psychometric properties")
		AND
**3**	Subject headings	Interpersonal violence/crime
	Keywords	(Violen* OR aggress* OR assault*)
		AND
**4**	Subject headings	Discharge
	Keywords	("Post discharge" OR discharge OR community OR psychiatri* OR forensic* OR secur* OR release* OR custod*)

Table S3. Risk assessment instruments used to predict post-discharge violence

	Instrument	Abbreviation	Authors
1	Brief Assessment for Recidivism Risk	BARR-2002R	Babchishin et al. (2013)[21]
2	Comprehensive Assessment of Psychopathic Personality	CAPP	Cooke et al. (2004)[22]
3	Dynamic Risk Outcome Scales	DROS	Drieschner & Hesper (2008)[23]
4	Female Additional Manual	FAM	De Vogel et al. (2014)[24]
5	Forensic Psychiatry & Violence Oxford	FoVOx	Wolf et al. (2018)[25]
6	Historical Clinical Risk Management-20 version 2	HCR-20v2	Webster et al. (1995)[26]
7	Historical Clinical Risk Management-20 version 3	HCR-20v3	Douglas et al. (2013)[27]
8	Historical 10 (subscale of HCR-20)	H10	Webster et al. (1995)[26]
9	Historische Klinische, Toekomstige-20	HKT-30	Werkgroep Pilotstudy Risicotaxatie Forensische Psychiatrie (2002)[28]
10	Historische Klinische, Toekomstige Revisie	HKT-R	Spreen et al. (2014)[29]
11	Life-time Aggression	LHA	Brown et al. (1982)[30]
12	Level of Service/Risk–Need–Responsivity	LS/RNR	Andrews et al. (2008)[31]
13	Level of Service Inventory Revised	LSI-R:SV	Andrews & Bonta (2001)[32]
14	Medium Security Recidivism Guide	MSRAG	Hickey et al. (2009)[33]
15	Offender Group Reconviction Scale 3	OGRS	Howard et al. (2009)[34]
16	PosLHitive and Negative Syndrome Scale	PANSS	Kay et al. (1987)[35]
17	Psychopathy Checklist Screening Version	PCL:SV	Hart et al. (1995)[36]
18	Psychopathy Checklist Revised	PCL-R	Hare (1991)[37]
19	Psychopathic Personality Inventory	PPI	Lilenfield & Andrews (1996)[38]
20	Pencil and Paper Instrument for Violence	PIV	Coid et al. (2016)[39]
21	Risk Assessment for Sex Offender Recidivism	RRASOR	Hanson (1997)[40]
22	Risk Matrix 2000	RM2000	Thornton (2007)[41]
23	Structured Anchored Clinical Judgement (Minimum Set)	SACJ-Min	Grubin (1998)[42]
24	Structured Assessment of Protective Factors	SAPROF	de Vogel et al. (2009)[43]
25	Structured Assessment of Violence Risk in Youth	SAVRY	Borum et al. (2006)[44]
26	Sex Offender Risk Appraisal Guide	SORAG	Quinsey et al. (2006)[45]
27	Short-Term Assessment of Risk and Treatability	START	Webster et al. (2004)[46]
28	Static 2002	Static 2002	Hanson & Thornton (2003)[47]
29	Static 2002 Revised	Static 2002R	Helmus et al. (2012)[48]
30	Static-99	Static 99	Hanson & Thornton (2000)[49]
31	Static 99 Revised	Static 99R	Helmus et al. (2012) [48]
32	Sexual Violence Risk - 20	SVR-20	Boer et al. (1997)[50]
33	Violence Risk Appraisal Guide	VRAG	Harris et al. (1993)[51]
34	Violence Risk Screening-10	VRAG-R	Bjørkly et al. (2009)[52]
35	Violence Risk Scale	VRS	Wong & Gordon (2000)[53]
36	Violence Risk Scale Sexual Offence	VRS-SO	Wong et al. (2003-2007)[54]

Table S4. Study characteristics table

Author, date and location	Study design	Study period	Multi-centre	Risk assessment instrument	Sample size	Study sample	% Male	Age	Diagnosis (diagnoses)	Outcome	Follow-up period	Type of instrument evaluation
Barber-Rioja et al. (2012)[55]<br><br>*USA*	Prospective cohort	2006 - 2010	Yes	HCR-20v2; PCL:SV	131	Criminal defendants arrested for a misdemeanour or felony charges who chose mental health diversion as opposed to incarceration	68·7	37·3	Major depressive disorder (30%), bipolar (26%), schizophrenia (19%), anxiety-related disorder (8%), delusion disorder (8%), schizoaffective disorder (6%), other (3%)	Recidivism or violation of the conditions of a plea agreement	12 months	External
Bengtson et al. (2008)[56]<br><br>*Denmark*	Retrospective cohort	1978 - 1992	Yes	Static-99; Static-2002; Risk Matrix 2000	304	Adult males suspected of having committed a sexual offence who underwent a pre-trail FPE	100	32·7	Non-psychotic psychiatric disorder (96%), e.g., personality-disordered, developmentally disabled, mildly retarded etc	Violent and sexual recidivism	Mean period of 16·2 years	External
Bogaerts et al. (2018)[57]<br><br>*Netherlands*	Retrospective cohort	2004 - 2011	Yes	HKT-R	347	All Dutch forensic psychiatric patients discharged between 2004-2008	91·9	40	Personality disorders NOS (52%), substance-related disorders (33%), cluster B personality disorders (28%), schizophrenia and psychotic disorders (21%)	Violent recidivism	2 and 5 years	External
Brookstein et al. (2020)[58]<br><br>*Australia*	Retrospective cohort	2000 - 2013	No	HCR-20v2; HCR-20v3	100	Adult forensic psychiatric patients whose files were retrieved from archives of comparative datasets	73	33·5	Schizophrenia spectrum and other psychotic disorders (72%), depressive disorders (8%), bipolar and related disorders (6%), substance related disorders (17%), trauma and stress-	Violent recidivism	Maximum follow up period of 12 years and 10 months	External

									related disorders (5%), OCD (1%), eating disorders (1%)			
Brouillette-Alarie & Proulx (2013)[59]<br><br>*Canada*	Retrospective cohort	1979 - 2006	No	Static-99; Static-99R	228	Adult male sex offenders discharged a maximum-security psychiatric facility	100	40·9		Violent recidivism	5 years	Internal
Canales et al. (2014)[60]<br><br>*Canada*	Retrospective cohort	2000 - 2008	No	LS/RNR	138	Community-supervised mentally disordered offenders	71·7	35·5	Psychosis (41%), mood/anxiety disorders (31%), personality disorder (32%), cognitive dysfunction (21%), dual diagnosis (38%)	Violent and general recidivism	Mean period of 4 (2·1) years, range of 0·3 to 8 years	External
Coid et al. (2016)[39]<br><br>*England and Wales*	Prospective cohort	2010 - 2011	Yes	MSRAG; HCR-20v3; SAPROF; PCL:SV; PANSS; RM2000; OGRS2; PIV	387	Forensic psychiatric patients discharged from medium and low secure units into the community	89·2	37·6	Schizophrenia (65%), schizoaffective disorder (13%), mania/bipolar (7%), anxiety disorder (1%), personality disorder (2%), depression (1%), other (6%), no diagnosis (5%)	Any violence after discharge; general/any recidivism	6 and 12 months	External
Delforterie et al. (2020)[61]<br><br>*Netherlands*	Prospective	2007 - 2014	No	DROS; HKT-30	236	Forensic patients with a mild intellectual disability (mid) or borderline intellectual functioning (bif)	94·4	36·4	Mild intellectual disability or borderline intellectual functioning (mid-bif) (100%), substance use disorder (60%), personality disorder (53%), psychotic disorder (23%)	Violent, general and sexual recidivism	Mean period of 81 months	External

de Vogel et al. (2019)[62]<br><br>*Netherlands*	Retrospective cohort	1993 - 2016	Yes	HCR-20; HCR-20v3; FAM;<br><br>START; SAPROF; PCL-R	71	Female forensic psychiatric patients admitted and discharged from multiple facilities	0	38·6	Substance abuse (65%), borderline personality disorder (51%)	Violent and all recidivism	3 (pre-set) and 11·8 (mean) years	External
de Vogel et al. (2004)[63]<br><br>*Netherlands*	Retrospective cohort	1977 - 2001	No	SVR-20; Static-99	122	Forensic psychiatric patients admitted under tbs-order	100	24·8	Alcohol abuse (36%), drug abuse (3%), multiple substance abuse (20%)	Violent, general and sexual recidivism	Mean period of 140 months, range 20-291 months	External
de Vogel et al. (2014)[18]<br><br>*Netherlands*	Retrospective cohort	1990 - 2009	No	HCR-20v3	86	Male forensic psychiatric patients convicted of non-sexual violent offences and discharged from hospital	100	32·.1	Personality disorder (63%), psychotic disorder, (21%), inconclusive (16%)	Violent recidivism	1, 2, and 3 years	Development
de Vries Robbe et al. (2013)[64]<br><br>*Netherlands*	Retrospective cohort	1984 - 2009	No	HCR-20v2; SAPROF	188	Male forensic psychiatric patients with a history of violent/sexual violent offending	100	32	Axis II personality disorders (66%), Axis II personality disorder traits (22%), psychotic disorders (15%), sexual disorders, substance abuse disorders, mood disorders, other	Violent recidivism	1 and 3 years, and long term (mean of 11·1 years)	External
de Vries Robbe et al. (2015)[65]<br><br>*Netherlands*	Retrospective cohort	1984 - 2006	Yes	HCR-20v2; SAPROF; SVR-20	83	Male sexual offenders admitted to two different Dutch forensic psychiatric hospitals	100	30	Axis II personality disorders (5%) or traits (29%), psychotic disorder (3%), sexual disorder (14%)	General and sexual recidivism	1 year (violence only), 3 years, and long term (M = 15·1 years)	External
Dolan & Khawaja (2004)[66]<br><br>*England and Wales*	Retrospective cohort	1992 - 2000	No	HCR-20v2	70	Male forensic psychiatric patients with a history of violent offending who were discharged to the community	100	35·3	Schizophrenia (73%), organic brain syndromes (24%), schizoaffective disorder (7%), affective disorders (1%), personality disorder (31%),	Violent recidivism	Minimum 2 years	External

									substance misuse (44%)			
Douglas et al. (2003)[67] *Canada*	Retrospective cohort	1996 - 2001	No	HCR-20v2	100	Forensic psychiatric patients released from dispositions of not guilty by reason of insanity	91	35·3	Schizophrenia (74%), mood disorders (18%), substance abuse (5%), personality disorders (18%), other (3%)	Post-release violence	Median Period of 45·3 months, range 0·1 to 63·1 month	External
Ducro & Pham (2006)[68] *Belgium*	Retrospective cohort	1982 - 2003	No	SORAG; Static-99	147	Male forensic psychiatric patients who had been convicted of sex offences	100	45	Schizophrenia (13%), personality disorder (30%)	Violent and general and sexual recidivism	Mean period of 4·2 years	External
Eher et al. (2020)[69] *Austria*	Retrospective cohort	2008 - 2015	Yes	VRS-SO; Static-99	85	Male sexual offenders in Austria previously placed in a psychiatric facility due to a criminal court decision and released between 2008 - 2012	100	53·3	Personality disorder 93%), paraphilic disorder (76%), psychotic disorders (5%), mood disorder (10%), anxiety disorder (4%)	Sexual recidivism	Mean period of 7·2 years	External
Ferguson et al. (2009)[70] *Australia*	Retrospective cohort		No	LSI-R:SV	208	Forensic psychiatric patients discharged from a forensic psychiatric facility	75·5	30·8	Schizophrenia or psychotic disorder (67%)	Violent and any recidivism		External
Gammelgard et al. (2015)[71] *Finland*	Prospective cohort	2003 - 2012	No	SAVRY	65	Adolescent forensic psychiatric patients discharged from an inpatient unit for disruptive young people	68	22·3	Schizophrenia, schizotypal, delusional, and other non-mood psychotic disorders (25%), personality disorder and behavioural emotional disorders (49%), other (26%)	Violent and any crime	4 years	External

Gonsalves et al. (2013)[72] *USA*	Prospective cohort		No	PPI; PCL-R	83	Male forensic psychiatric patients discharged from a state forensic psychiatric hospital	100	33·9		Violent and sexual recidivism		External
Grann et al. (2000)[73] *Sweden*	Retrospective cohort	1988 - 1995	Yes	H-10; VRAG	404	All violent offenders from 1989 to 1990 diagnosed with personality disorder with or without concomitant abuse/dependence of alcohol or drugs and all male violent offenders with schizophrenia between 1988 and 1993 who were subjected to court ordered FPE in Sweden	91.1		Schizophrenia (27%), personality disorder (73%)	Violent recidivism	2 years	External
Gray et al. (2007)[74] *United Kingdom*	Pseudoprospective cohort	1990 - 2001	Yes	VRAG; PCL:SV; HCR-20v2; OGRS/OGRS2[a]	145 (ID)[aa] 996 (non-ID)	Forensic psychiatric patients discharged from four independent sector hospitals in the UK, separated into intellectual disability (ID) and non-ID groups	81·4 (ID) 85·6 (non-ID)	31.5	Schizophrenia (48%), personality disorders (19%), affective disorders (10%), organic mental disorders (28%), mental and behavioural disorders due to psychoactive substance use (10%), mental impairment (10%), neurotic (2%)	Violent and general recidivism	5 years	External
Harris et al. (2002)[75] *Canada*	Prospective cohort	1990 - 1998	Yes	VRAG	133	Insanity acquitees, persons found unfit to stand trial, plus all other persons occupying beds in secure	100		Violent offenders: psychotic disorder (72%), personality disorder (25%);	Violent recidivism	5 years	External

						psychiatric units in Ontario, June 1990			Non-violent: psychotic disorder (86%), personality disorder (8%)			
Hanson & Thornton (2000)[49]<br><br>*Canada*	Retrospective cohort	1972 - 1994	Yes	Static-99; RRASOR; SACJ-Min	344 (IPP)<br><br>142 (Oak Ridge)	Sexual offenders treated at two maximum-security psychiatric facilities / mental health centres		36·2		Violent and sexual recidivism	4 years (PPI)<br>10 years (Oak Ridge)	Development (Static-99), internal (RRASOR), and external (SACJ-Min)
Hildebrand et al. (2004)[76]<br><br>*Netherlands*	Retrospective cohort	1975 - 2001	No	PCL-R	94	Convicted rapists involuntarily admitted to a forensic psychiatric hospital, under TBS between 1975 and 1996	100	24·5		Violent, general and sexual recidivism	Mean period of 11·8 years	External
Ho et al. (2009)[77]<br><br>*United Kingdom*	Retrospective cohort	2001 - 2006	No	H10; PCL:SV; VRAG	96	Forensic psychiatric patients discharged from the Orchard Clinic in-patient psychiatric unit between December 2001 and April 2004	91·7	34·2	Schizophrenia or schizoaffective disorder (70.8%), bipolar affective disorder (16.7%), delusional disorder (6.25%), depression (4.17%)	Serious violent convictions	2 years	External
Hogan & Olver (2019)[78]<br><br>*Canada*	Retrospective cohort	2005 - 2017	No	HCR-20v3; PCL-R; START; VRS, VRAG-R (pre-treatment scores)	82	Forensic psychiatric patients discharged from a maximum-security hospital	93·9	37·7	Schizophrenia (76%), alcohol-related disorder (68%)	Violent and general recidivism	Mean period of 8·2 years	External
Jeandarme et al. (2017)[79]<br><br>*Belgium*	Retrospective cohort			PCL-R;	162	Patients found not guilty by reason of insanity who were transferred from prison to a medium-security hospital	98·7	35·3	Substance-related disorders (59%), psychotic disorders (38%), other disorders (25%), no disorders (8%), paraphilia (4%), panic disorders (2%), mood	Violent and general recidivism	2 years	External

									disorders (6%), developmental disorders (6%)			
Jeandarme et al. (2016)[80] *Belgium*	Prospective cohort	2001 - 2013	Yes	HCR-20v2	105	Forensic psychiatry patients admitted by not guilty by reason of insanity to three forensic medium secure units in Flanders	91·7	36·1	Personality disorders (76%), substance use disorders (61%), psychotic disorders (43%)	Violent recidivism	Mean period of 2 year	External
Jung et al. (2018)[81] *Canada*	Retrospective cohort		Yes	Static-2002R; BARR-2002R; Static-99R; SORAG	342[b]	Male sexual offender patient files gathered from outpatient forensic psychiatric clinic and an affiliated secure forensic psychiatric hospital	100	39·4		Violence, sexual and any recidivism	Mean period of 5·8 years	External
Kikuchi et al. (2021)[82] *Japan*	Prospective cohort		No	START	181	Forensic psychiatric patients given a 'Medical Treatment and Supervision Act' outpatient treatment order by the district court and were dwelling in the community	79	42·8	Schizophrenia, schizotypal and delusional disorders (78%), mental and behavioural disorders due to psychoactive and other substance use (11%), mood or affective disorders (5%), neurotic, stress-related and somatoform disorders (2%), organic and symptomatic mental disorders (1%), mental retardation (1%), disorders of psychological development (1%), epilepsy and	Physical violence	6 months	External

									recurrent seizures (1%), disorders of adult personality and behaviour (1%)			
Michel et al. (2013)[83]<br><br>*Canada, Finland, Germany, Sweden*	Prospective cohort		Yes	HCR-20v2	150	Male patients with schizophrenia discharged from forensic hospitals across four sites	100	40	Schizophrenia (83%), schizoaffective (17%), antisocial personality disorder (29%), diagnosis of alcohol abuse or dependence (59%), diagnosis of drug abuse or dependence (46%)	Aggressive behaviour	6, 12, 18 and 24 months	External
Nicholls (2004)[84]<br><br>*Canada*	Retrospective cohort	1992 - 2000	No	HCR-20v2; PCL:SV	70 (HCR20)<br><br>77 (PCL:SV)	Women found to be not criminally responsible on account of mental disorder and discharged; men were matched to women by date	50	34·6	Schizophrenia (26%), schizoaffective (9%), delusional disorder (2%), depressive disorder (9%), bipolar disorder (21%), drug induced psychosis (9%), mental retardation (4%), borderline IQ (4%), schizoid (2%), antisocial (2%), borderline (9%), mixed NOS, no diagnosis, other[c] (15%)	Physical aggression and general recidivism	Mean period of 3·8 years	External
Nilsson et al. (2011)[85]<br><br>*Sweden*	Prospective cohort	1998 - 2005	No	HCR-20v2; PCL-R; LHA	99	Violent and/or sexually violent offenders who underwent a pre-trial FPE and subsequently found guilty	92	30	Psychosis (20%), personality disorder (67%), antisocial personality disorder (42%), substance abuse / dependence (53%), conduct	Violent recidivism	Mean period of 59·3 months	External

									disorder during childhood (48%)			
Nowak & Nugter (2014)[20]<br>*Netherlands*	Retrospective cohort		Yes	HKT-30; PCL-R	69	Forensic psychiatric patients under compulsory treatment with psychotic disorders		36	Psychotic disorder (100%)	General recidivism	100 months	External
Pedersen et al. (2010)[86]<br>*Denmark*	Retrospective cohort	2001 - 2007	No	PCL:SV; CAPP; HCR-20v2[d]	96	Patients discharged from a forensic psychiatric unit	100	36	Schizophrenia (75%), affective disorders (9%), personality disorders (20%), other diagnoses (4%), substance use disorder (44%)	Violent and non-violent crime	Mean period of 5·7 years	External
Pedersen et al. (2012)[87]<br>*Denmark*	Prospective cohort	2006 - 2008	No	HCR-20v2	81	Patients discharged from a forensic psychiatric unit	100	35·7	Schizophrenia (79%), affective disorders (3%), personality disorders (9%), other (6%)	Violent recidivism	Mean period of 21 months	External
Pham & Ducro (2008)[19]<br>*Belgium*	Prospective cohort		No	SORAG; Static-99	133 (SORAG)<br>137<br>(Static-99)	Forensic psychiatric patients arrested for sexual offences and committed to a secure psychiatric hospital				Violent, general and sexual recidivism		External
Schaap et al. (2009)[88]<br>*Netherlands*	Quasi-prospective	1985 - 2001	Yes	HCR-20v2; PCL-R	45	Female forensic psychiatric patients under TBS-order	0	28·3		Violent and general recidivism		External
Shepherd et al. (2018)[89]<br>*Australia*	Retrospective cohort	2001 - 2006	No	HCR-20v2	116	Randomly selected male and female forensic psychiatric patients,	72	32·2	Psychotic or affective disorders (72%)	Any violent and any recidivism	Mean period of 4·4 years	External

						discharged from a secure forensic hospital						
Sjöstedt & Långström (2002)[90]<br><br>*Sweden*	Retrospective cohort	1988 - 1999		PCL-R; SVR-20; VRAG; RRASOR	51	Male offenders diagnosed with a personality disorder, convicted of attempted or completed rape and subjected to court-ordered FPE during 1988 - 1990	100	34	Personality disorder (100%)	Violent and sexual recidivism	Mean period of 92·3 months	External
Stadtland et al. (2005)[91]<br><br>*Germany*	Prospective cohort	1992 - 2002	Yes	PCL-R	71	Offenders assessed for their criminal responsibility who met ICD-10 criteria for major mental disorders	83·6	34·6	Organic psychosis, schizophrenia, mood disorders, adjustment disorders, mental retardation, severe attention deficit disorders	Violent and non-violent recidivism	Mean period of 58·6 months	External
Tengstrom (2001)[92]<br><br>*Sweden*	Retrospective cohort	1988 - 1998		VRAG; H-10;	106	Male violent offenders referred for the first time to a court-ordered pre-sentence FPE	100	33	Schizophrenia (100%)	Violent recidivism	Minimum 5 years, mean period 86 months	External
Tengstrom et al. (2000)[93]<br><br>*Sweden*	Retrospective cohort	1988 - 1996		PCL-R	141	Male violent offenders referred for the first time to a court-ordered pre-sentence FPE	100	33	Schizophrenia (81%), schizoaffective disorder (7%), other psychoses (12%)	Violent recidivism	Mean period of 51 months, follow-up for 1, 2, 3, 4 and 5 years	External
Thomson et al. (2008)[94]<br><br>*United Kingdom*	Prospective cohort	1992 - 2001	No	H-10; VRAG, PCL-R	54	Forensic psychiatric patients with schizophrenia resident at a high security State Hospital	90	35·5	Schizophrenia (100%), antisocial personality disorder (32%), alcohol dependence (26%), drug dependence	Violent and general recidivism	Mean period of 8·7 years	External

									(39%), mental handicap (4%)			
Troquete et al. (2015)[95]<br><br>*Netherlands*	Randomised controlled trial	2007 - 2010	Yes	START; H10	163	Case managers enrolled in the RACE trial recruited their outpatient forensic psychiatry patients	94	40	Personality disorders (69%), substance-related disorders (38%), impulse control disorders (27%), mood disorders (21%), paraphilia (205), psychotic disorder (7%), no diagnosis on Axis I (7%)	Violent or criminal behaviour	3 and 6 months	External
van Heesch et al. (2016)[96]<br><br>*Belgium*	Retrospective cohort	2001 - 2010	No	VRAG	191	Male forensic psychiatric patients admitted to a medium secure unit in Flanders between 2001 and 2010	100	37·2	Personality disorders (75%), substance-related disorders (60%), psychotic disorders (45%), intellectual disability (14%)	Violent recidivism	Mean period of 2·44 years	External
Vojt et al. (2013)[97]<br><br>*United Kingdom*	Prospective cohort	2005 – 2010	No	HCR-20v2	66	Mentally disordered offenders discharged from the State Hospital (a high secure psychiatric hospital)	100	38·6	Schizophrenia (85%), Personality disorders (7%)	Violent conviction	Mean Period of 31 months	External
Wallinius et al. (2012)[98]<br><br>*Sweden*	Prospective cohort	1992 [52]-	Yes	PCL-R	153	All male patients undergoing FPE at the state units	100	Got= 35; Upp = 34	Mood disorders (45%; 20%), 25% (21%) anxiety disorders (25%; 21%), psychotic disorders (0%; 17%), substance use disorders (50%; 61%), cluster A personality disorders (37%; 15%), cluster B personality disorders (53%;	Violent recidivism and general criminality	Mean period of 6 - 8 years (Gothenburg)<br><br>Mena period of 4 – 6 years (Uppsala)	External

									61%), cluster C personality disorders (26%; 20%), antisocial disorders (44%; 33%), borderline personality disorder (30%; 28%), conduct disorder (48%; 44%)[e]			
Wolf et al. (2018)[25]<br><br>*Sweden*	Retrospective cohort	1992 - 2013	Yes	FoVOx	2248	All patients discharged from secure hospitals in Sweden between January 1992 and December 2013	86	36	Schizophrenia-spectrum (46%), bipolar (6%), unipolar depression (5%), anxiety disorders (7%), other (37%)	Violent recidivism	12 and 24 months	Development and internal

Note. [a] data extracted from duplicate paper Snowden et al. (2007)[70]. [aa] This review only analyses data from the non-ID sample, excluding the ID sample, included in Gray et al. (2007).[44]

[b] sample size varied by risk assessment instrument: BARR-2002R (n = 342); Static-2002R (n = 339); Static-99R (n = 310); SORAG (n = 113)

[c] % diagnosis for male patients extracted

[d] data extracted from salami paper Pedersen, Rasmussen & Elsass (2010)[71]

[e] % given in brackets related to diagnoses of each sample, e.g., (Gothenburg%; Uppsala%)

Table S5. Quality assessment (risk of bias) ratings for reviewed studies

Study	PROBAST Risk of Bias Judgement					Study Applicability
	Participants	Predictors	Outcome	Analysis	Overall	
Barber-Rioja et al. (2012)[55]	Low	High	Low	High	High	High
Bengtson et al. (2008)[56]	Low	Low	Low	High	High	Low
Bogaerts et al. (2018)[57]	Low	Low	Low	High	High	Low
Brookstein et al. (2020)[58]	Low	Low	Low	High	High	Low
Brouillette-Alarie & Proulx (2013)[59]	Low	Low	Low	High	High	Unclear
Canales et al. (2014)[60]	Low	Low	Low	High	High	Low
Coid et al. (2016)[39]	Low	Low	Low	High	High	Low
Delforterie et al. (2020)[61]	Low	Low	Low	High	High	Low
de Vogel et al. (2019)[62]	Low	Low	Low	High	High	Low
de Vogel et al. (2004)[63]	Low	Low	Low	High	High	Low
de Vogel et al. (2014)[18]	Low	Low	Unclear	High	High	Low
de Vries Robbe et al. (2013)[64]	Low	Low	Low	High	High	Low
de Vries Robbe et al. (2015)[65]	Low	Low	Low	High	High	Low
Dolan & Khawaja (2004)[66]	Low	Low	Low	High	High	Low
Douglas et al. (2003)[67]	Low	Low	Low	High	High	Low
Ducro & Pham (2006)[68]	Low	High	Low	High	High	High
Eher et al. (2020)[69]	Low	Low	Low	High	High	High
Ferguson et al. (2009)[70]	Low	Low	High	High	High	Low
Gammelgard et al. (2015)[71]	Low	Low	Low	High	High	Low
Gonsalves et al. (2013)[72]	Low	Low	High	High	High	Low
Grann et al. (2000)[73]	Low	Low	Unclear	High	High	Low
Gray et al. (2007)[74]	Low	Low	Low	High	High	Low
Harris et al. (2002)[75]	High	Low	Low	High	High	Low
Hanson & Thornton (2000)[49]	Low	High	Low	High	High	High
Hildebrand et al. (2004)[76]	Low	Low	Low	High	High	Low
Ho et al. (2009)[77]	Low	Low	Low	High	High	
Hogan & Olver (2019)[78]	Low	Low	Low	High	High	Low
Jeandarme et al. (2017)[79]	Low	Unclear	Low	High	High	Low
Jeandarme et al. (2016)[80]	Low	Low	Low	High	High	Low
Jung et al. (2018)[81]	Low	Low	Low	High	High	Unclear
Kikuchi et al. (2021)[82]	Low	Low	High	High	High	High
Michel et al. (2013)[83]	Low	Low	Low	High	High	Low
Nicholls (2004)[84]	Low	Low	Low	High	High	Low
Nilsson et al. (2011)[85]	Low	Low	Low	High	High	Low
Nowak & Nugter (2014)[20]	Unclear	Low	Low	High	High	Low
Pedersen et al. (2010)[86]	Low	Low	Low	High	High	Low
Pedersen et al. (2012)[87]	Low	Low	Low	High	High	Low
Pham & Ducro (2008)[19]	Low	Unclear	High	High	High	Unclear
Schaap et al. (2009)[88]	Low	High	High	High	High	High
Shepherd et al. (2018)[89]	Low	Low	Low	High	High	Low

Sjostedt & Langstrom (2002)[90]	Low	Low	Low	High	High	Low
Stadtland et al. (2005)[91]	Low	Low	Low	High	High	Low
Tengstrom (2001)[92]	Low	Low	Low	High	High	Low
Tengstrom et al. (2000)[93]	Low	Low	Low	High	High	Low
Troquete et al. (2015)[94]	Low	Low	Low	High	High	Low
Thomson et al. (2008)[95]	Low	Low	Low	High	High	Low
van Heesch et al. (2016)[96]	Low	Low	Low	High	High	Low
Vojt et al. (2013)[97]	Low	Low	Low	High	High	Low
Wallinius et al. (2012)[98]	Low	Low	Low	High	High	Low
Wolf et al. (2018)[25]	Low	Low	Low	Low	Low	Low

Supplementary References

1 Singh JP, Desmarais SL, Van Dorn RA. Measurement of predictive validity in violence risk assessment studies: A second-order systematic review. *Behav Sci Law* 2013; **31**: 55–73.

2 Hajian-Tilaki KO, Hanley JA. Comparison of three methods for estimating the standard error of the area under the curve in ROC analysis of quantitative data. *Acad Radiol* 2002; **9**: 1278–85.

3 Hanley JA, McNeil BJ. The meaning and use of the area under a receiver operating characteristic (ROC) curve. *Radiology* 1982; **143**: 29–36.

4 Strand S, Belfrage H, Fransson G, Levander S. Clinical and risk management factors in risk prediction of mentally disordered offenders-more important than historical data?: A retrospective study of 40 mentally disordered offenders assessed with the HCR-20 violence risk assessment scheme. *Legal and Criminol Psychol* 1999; **4**: 67–76.

5 De Vries Robbé M, de Vogel V, Douglas KS, Nijman HLI. Changes in dynamic risk and protective factors for violence during inpatient forensic psychiatric treatment: Predicting reductions in postdischarge community recidivism. *Law Hum Behav* 2015; **39**: 53–61.

6 Snell KI, Ensor J, Debray TP, Moons KG, Riley RD. Meta-analysis of prediction model performance across multiple studies: Which scale helps ensure between-study normality for the C-statistic and calibration measures? *Stat Methods Med Res* 2018; **27**: 3505–22.

7 Viechtbauer W. Bias and efficiency of meta-analytic variance estimators in the random-effects model. *J Educ Behav Stat* 2005; **30**: 261–93.

8 Knapp G, Hartung J. Improved tests for a random effects meta-regression with a single covariate. *Stat Med* 2003; **22**: 2693–710.

9 RStudio Team. RStudio: Integrated Development for R. 2020. http://www.rstudio.com/.

10 Viechtbauer W. Conducting meta-analyses in R with the metafor package. *J Stat Softw* 2010; **36**: 1–48.

11 Balduzzi S, Rücker G, Schwarzer G. How to perform a meta-analysis with R: a practical tutorial. *Evid Based Ment Health* 2019; **22**: 153–60.

12 Schwarzer G. meta: An R package for meta-analysis. *R news* 2007; **7**: 40–5.

13 Harrer M, Cuijpers P, Furukawa T, Ebert DD. dmetar: Companion R package for the guide 'Doing meta-analysis in R'. 2019. Avaliable from: http://dmetar.protectlab.org/

14 McGuinness LA, Higgins JP. Risk-of-bias VISualization (robvis): an R package and Shiny web app for visualizing risk-of-bias assessments. *Res Synth Methods* 2021; **12**: 55–61.

15 Ban J-W, Emparanza JI, Urreta I, Burls A. Design Characteristics Influence Performance of Clinical Prediction Rules in Validation: A Meta-Epidemiological Study. *PLOS One* 2016; **11**: e0145779.

16 Debray TP, Damen JA, Snell KI, *et al.* A guide to systematic review and meta-analysis of prediction model performance. *BMJ* 2017; **356**: i6460.

17 Moons KGM, Groot JAH de, Bouwmeester W, et al. Critical Appraisal and Data Extraction for Systematic Reviews of Prediction Modelling Studies: The CHARMS Checklist. *PLOS Medicine* 2014; **11**: e1001744.

18 de Vogel V, van den Broek E, de Vries Robbe M. The use of the HCR-20V3 in Dutch forensic psychiatric practice. *Int J Forensic Ment Health* 2014; **13**: 109–21.

19 Pham T, Ducro C. Risk assessment in social defence: Preliminary factorial analysis of the SORAG 'Sex Offender Recidivism Appraisal Guide' (SORAG) and the Static-99. *Ann Med Psychol* 2008; **166**: 575–9.

20 Nowak M, Nugter M. 'I'll serve my time...' II. A replication study of recidivism in patients with a one-year hospital order. *Tijdschr Psychiatr* 2014; **56**: 237–46.

21 Babchishin KM, Hanson RK, Blais J. User Guide for the Brief Assessment for Recidivism Risk – 2002R. 2013.

22 Cooke, D. J., Hart, S. D., Logan, C., Micheie, C. Comprehensive Assessment of Psychopathic Personality – Institutional Rating Scale (CAPP IRS). 2004.

23 Drieschner KH, Hesper BL. Dynamic risk outcome scales. *Boschoord: Trajectum Hoeve Boschoord* 2008.

24 De Vogel V, de Vries Robbé M, Van Kalmthout W, Place C. Female Additional Manual (FAM). Additional guidelines to the HCR-20 for assessing risk for violence in women. *Utrecht, The Netherlands: Van der Hoeven Stichting* 2014.

25 Wolf A, Fanshawe TR, Sariaslan A, Cornish R, Larsson H, Fazel S. Prediction of violent crime on discharge from secure psychiatric hospitals: A clinical prediction rule (FoVOx). *Eur Psychiatry* 2018; **47**: 88–93.

26 Webster CD, Eaves D. The HCR-20 scheme: the assessment of dangerousness and risk. Vancouver, B.C.? Mental Health, Law and Policy Institute, Department of Psychology, Simon Fraser University and Forensic Psychiatric Services Commission of British Columbia.

27 Douglas KS, Hart SD, Webster CD, Belfrage H. HCR-20v3 : assessing risk for violence : user guide. Burnaby, British Columbia: Mental Health, Law and Policy Institute, Simon Fraser University, 2013.

28 Werkgroep Pilotstudy Risicotaxatie Forensische Psychiatrie. Bevindingen Van Een Landelijke Pilotstudy Naar de HKT-30 [Findings of a Nationwide Pilot Study on the HKT-30]. The Hague: Ministerie van Justitie, 2002.

29 Spreen, Marinus, Brand, Eddy, Ter Horst, Paul, Bogaerts, Stefan, Developmental Psychology. Handleiding en Methodologische Verantwoording HKT-R, Historisch, Klinische en Toekomstige - Revisie. Dr. van Mesdag kliniek, 2014 https://research.tilburguniversity.edu/en/publications/51ca49c1-550c-4fe1-8c41-b0cbfbfc5839 (accessed June 1, 2023).

30 Brown GL, Elber MH, Goyer PF, Jimerson DC, Klein WJ et al. Aggression, suicide, and serotonin: relationships to CSF amine metabolites. *Am J Psychiatr* 1982, **139**: 741—746.

31 Andrews D, Bonta J, Wormith S. The Level of Service/Risk Need Responsivity Inventory (LS/RNR): Scoring Guide. Toronto, Ontario, Canada: Multi-Health Systems, 2008.

32 Andrews DA, Bonta J. The level of service inventory-revised : user's manual. North Tonawanda, New York: Multi-Health Systems, 2001.

33 Hickey N, Yang M, Coid J. The development of the Medium Security Recidivism Assessment Guide (MSRAG): an actuarial risk prediction instrument. *J Forensic Psychiatry & Psychol* 2009; **20**: 202–24.

34 Howard P, Francis B, Soothill K, Humphreys L. OGRS 3: the revised Offender Group Reconviction Scale. (Research Summary 7/09). Ministry of Justice.

35 Kay SR, Fiszbein A, Opler LA. The Positive and Negative Syndrome Scale (PANSS) for Schizophrenia. *Schizophr Bull* 1987; **13**: 261–76.

36 Hart SD, Cox D N, Hare RD. The Hare Psychopathy Checklist : Screening Version (PCL:SV). North Tonawanda, NY: Multi-Health Systems, Inc., 1995.

37 Hare RD. The Hare psychopathy checklist-revised : manual. North Tonawanda, N.Y.: Multi-Health Systems, Inc., 1991.

38 Lilienfeld SO, Andrews BP. Development and Preliminary Validation of a Self-Report Measure of Psychopathic Personality Traits in Noncriminal Population. *J Pers Assess* 1996; **66**: 488–524.

39 Coid JW, Ullrich S, Kallis C, *et al*. Improving risk management for violence in mental health services: a multimethods approach. *PGfAR* 2016; **16**: 11.

40 Hanson, RK. The development of a brief actuarial risk scale for sexual offence recidivism (User Report 97-04). Ottowa: Department of the Solicitors General of Canada; 1997

41 Thornton D. Scoring guide for risk matrix 2000.9/SVC. *Unpublished document* 2007.

42 Grubin, D. Sex Offending Against Children: Understanding the Risk | Office of Justice Programs. London: Home Office: Police Research Paper Series 99, 1998 https://www.ojp.gov/ncjrs/virtual-library/abstracts/sex-offending-against-children-understanding-risk (accessed June 1, 2023).

43 Vogel V de, Ruiter C de, Bouman Y, Vries Robbé M. SAPROF: guidelines for the assessment of protective factors for violence risk.English version. Utrecht, Netherlands: Forum Educatief, 2009.

44 Borum, R, Bartel, P, Forth, A. Structured Assessment of Violence Risk in Youth (SAVRY). Florida: Psychological Assessment Resource, Inc, 2006.

45 Quinsey VL, Harris GT, Rice ME, Cormier CA. Violent offenders: Appraising and managing risk. Washington, DC: American Psychological Association, 2006.

46 Webster CD, Martin M-L, Brink J, Nicholls TL. START: Short-term assessment of risk and treatability. Hamilton, Ontario: St Joseph's Healthcare, 2004.

47 Hanson RK, Thornton D. Notes on the development of Static-2002. Solicitor General Canada, 2003.

48 Helmus L, Thornton D, Hanson RK, Babchishin KM. Improving the Predictive Accuracy of Static-99 and Static-2002 With Older Sex Offenders: Revised Age Weights. *Sex Abuse* 2012; **24**: 64–101.

49 Hanson RK, Thornton D. Improving risk assessments for sex offenders: A comparison of three actuarial scales. *Law Hum Behav* 2000; **24**: 119–36.

50 Boer DP, Hart S, Kropp PR, Webster CD. Sexual Risk Violence-20. Burnaby, BC: Simon Fraser University, Mental Health, Law, and Policy Institute, 1997.

51 Harris GT, Rice ME, Quinsey VL. Violent recidivism of mentally disordered offenders: The development of a statistical prediction instrument. *Crim Justice Behav* 1993; **20**: 315–35.

52 Bjørkly S, Hartvig P, Heggen F-A, Brauer H, Moger TA. Development of a brief screen for violence risk (V-RISK-10) in acute and general psychiatry: An introduction with emphasis on findings from a naturalistic test of interrater reliability. *Eur Psychiatry* 2009; **24**: 388–94.

53 Wong S, Gordon A. Violence risk scale (VRS). Saskatoon, Saskatchewan, 2000.

54 Wong, S., Olver, M. E., Nicholaichuk, T P., Gordon, A. The Violence Risk Scale: Sexual Offense version (VRS-SO). Regional Psychiatric Centre and University Saskatewan, 2003.

55 Barber-Rioja V, Dewey L, Kopelovich S, Kucharski L. The utility of the HCR-20 and PCL:SV in the prediction of diversion noncompliance and reincarceration in diversion programs. *Crim Justice Behav* 2012; **39**: 475–92.

56 Bengtson S. Is newer better? A cross-validation of the Static-2002 and the Risk Matrix 2000 in a Danish sample of sexual offenders. *Psychol. Crime Law* 2008; **14**: 85–106.

57 Bogaerts S, Spreen M, ter Horst P, Gerlsma C. Predictive validity of the HKT-R risk assessment tool: Two and 5-year violent recidivism in a nationwide sample of Dutch forensic psychiatric patients. *Int J Offender Ther Comp Criminol* 2018; **62**: 2259–70.

58 Brookstein DM, Daffern M, Ogloff JR, Campbell RE, Chu CM. Predictive validity of the hcr-20v3 in a sample of australian forensic psychiatric patients. *Psychiatry Psychol Law* 2020; **28**: 325–342.

59 Brouillette-Alarie S, Proulx J. Predictive validity of the Static-99R and its dimensions. *J Sex Aggress* 2013; **19**: 311–28.

60 Canales DD, Campbell MA, Wei R, Totten AE. Prediction of general and violent recidivism among mentally disordered adult offenders: Test of the Level of Service/Risk-Need-Responsivity (LS/RNR) Instrument. *Crim Justice Behav* 2014; **41**: 971–91.

61 Delforterie M, Hesper B, Nijman H, Korzilius H, Turhan A, Didden R. The predictive value of the Dynamic Risk Outcome Scales (DROS) for predicting recidivism in (forensic) patients with mild intellectual disabilities or borderline intellectual functioning. *Tijdschr Psychiatr* 2020; **62**: 1040–8.

62 de Vogel V, Bruggeman M, Lancel M. Gender-sensitive violence risk assessment: Predictive validity of six tools in female forensic psychiatric patients. *Crim Justice Behav* 2019; **46**: 528–49.

63 de Vogel V, de Ruiter C, van Beek D, Mead G. Predictive validity of the SVR-20 and Static-99 in a Dutch sample of treated sex offenders. *Law Hum Behav* 2004; **28**: 235–51.

64 de Vries Robbe M, de Vogel V, Douglas KS. Risk factors and protective factors: A two-sided dynamic approach to violence risk assessment. *J Forensic Psychiatry Psychol* 2013; **24**: 440–57.

65 de Vries Robbe M, de Vogel V, Koster K, Bogaerts S. Assessing protective factors for sexually violent offending with the SAPROF. *Sex. Abuse: J Res Treat* 2015; **27**: 51–70.

66 Dolan M, Khawaja A. The HCR-20 and post-discharge outcome in male patients discharged from medium security in the UK. *Aggress Behav* 2004; **30**: 469–83.

67 Douglas KS, Ogloff JR, Hart SD. Evaluation of a Model of Violence Risk Assessment Among Forensic Psychiatric Patients. *Psychiatr Serv* 2003; **54**: 1372–9.

68 Ducro C, Pham T. Evaluation of the SORAG and the Static-99 on Belgian Sex Offenders Committed to a Forensic Facility. *Sex. Abuse: J Res Treat* 2006; **18**: 15–26.

69 Eher R, Hofer S, Buchgeher A, Domany S, Turner D, Olver ME. The Predictive Properties of Psychiatric Diagnoses, Dynamic Risk and Dynamic Risk Change Assessed by the VRS-SO in Forensically Admitted and Released Sexual Offenders. *Front. Psychiatry* 2020; **10**: 922.

70 Ferguson AM, Ogloff JRP, Thomson L. Predicting recidivism by mentally disordered offenders using the LSI-R: SV. *Crim Justice Behav* 2009; **36**: 5–20.

71 Gammelgard M, Koivisto A-M, Eronen M, Kaltiala-Heino R. Predictive validity of the structured assessment of violence risk in youth: A 4-year follow-up. *Crim Behav Ment Health* 2015; **25**: 192–206.

72 Gonsalves VM, McLawsen JE, Huss MT, Scalora MJ. Factor structure and construct validity of the psychopathic personality inventory in a forensic sample. *Int J Law Psychiatry* 2013; **36**: 176–84.

73 Grann M, Belfrage H, Tengstrom A. Actuarial assessment of risk for violence: Predictive validity of the VRAG and the historical part of the HCR-20. *Crim Justice Behav* 2000; **27**: 97–114.

74 Gray NS, Fitzgerald S, Taylor J, MacCulloch MJ, Snowden RJ. Predicting future reconviction in offenders with intellectual disabilities: The predictive efficacy of VRAG, PCL-SV, and the HCR-20. *Psychol Assess* 2007; **19**: 474–9.

75 Harris GT, Rice ME, Cormier CA. Prospective replication of the Violence Risk Appraisal Guide in predicting violent recidivism among forensic patients. *Law Hum Behav* 2002; **26**: 377–94.

76 Hildebrand M, de Ruiter C, de Vogel V. Psychopathy and Sexual Deviance in Treated Rapists: Association With Sexual and Nonsexual Recidivism. *Sex. Abuse: J Res Treat* 2004; **16**: 1–24.

77 Ho H, Thomson L, Darjee R. Violence risk assessment: The use of the PCL-SV, HCR-20, and VRAG to predict violence in mentally disordered offenders discharged from a medium secure unit in Scotland. *J Forensic Psychiatry Psychol* 2009; **20**: 523–41.

78 Hogan NR, Olver ME. Static and dynamic assessment of violence risk among discharged forensic patients. *Crim Justice Behav*; **46**: 923–38.

79 Jeandarme I, Edens JF, Habets P, Bruckers L, Oei K, Bogaerts S. PCL-R field validity in prison and hospital settings. *Law Hum Behav* 2017; **41**: 29–43.

80 Jeandarme I, Pouls C, De Laender J, Oei T, Bogaerts S. Field validity of the HCR-20 in forensic medium security units in Flanders. *Psychol Crim Law* 2016; **23**: 305–22.

81 Jung S, Wielinga F, Ennis L. Can we keep it simple? Using the BARR-2002R with a community-based sex offender sample. *J Sex Aggress* 2018; **24**: 25–36.

82 Kikuchi A, Soshi T, Kono T, Koyama M, Fujii C. Validity of Short-Term Assessment of Risk and Treatability in the Japanese Forensic Probation Service. *Front Psychiatry* 2021; **12**: 645927.

83 Michel SF, Riaz M, Webster C, *et al.* Using the HCR-20 to predict aggressive behavior among men with schizophrenia living in the community: Accuracy of prediction, general and forensic settings, and dynamic risk factors. *Int J Forensic Ment Health* 2013; **12**: 1–13.

84 Nicholls TL. Violence risk assessments with female NCRMD acquittees: Validity of the HCR-20 and PCL-SV. *Diss Abstr B, Sci. eng* 2004; **64**: 4055.

85 Nilsson T, Wallinius M, Gustavson C, Anckarsater H, Kerekes N. Violent recidivism: a long-time follow-up study of mentally disordered offenders. *PLoS One* 2011; **6**: e25768.

86 Pedersen L, Kunz C, Rasmussen K, Elsass P. Psychopathy as a risk factor for violent recidivism: Investigating the Psychopathy Checklist Screening Version (PCL:SV) and the Comprehensive Assessment of Psychopathic Personality (CAPP) in a forensic psychiatric setting. *Int J Forensic Ment Health* 2010; **9**: 308–15.

87 Pedersen L, Ramussen K, Elsass P. HCR-20 violence risk assessments as a guide for treating and managing violence risk in a forensic psychiatric setting. *Psychol Crim Law* 2012; **18**: 733–43.

88 Schaap G, Lammers S, de Vogel V. Risk assessment in female forensic psychiatric patients: A quasi-prospective study into the validity of the HCR-20 and PCL-R. *J Forensic Psychiatry Psychol* 2009; **20**: 354–65.

89 Shepherd SM, Campbell RE, Ogloff JR. The utility of the HCR-20 in an Australian sample of forensic psychiatric patients. *Psychiatry Psychol Law* 2018; **25**: 273–82.

90 Sjostedt G, Langstrom N. Assessment of risk for criminal recidivism among rapists: A comparison of four different measures. *Psychol Crim Law* 2002; **8**: 25–40.

91 Stadtland C, Kleindienst N, Kroner C, Eidt M, Nedopil N. Psychopathic Traits and Risk of Criminal Recidivism in Offenders with and without Mental Disorders. *Int J Forensic Ment Health* 2005; **4**: 89–97.

92 Tengstrom A. Long-term predictive validity of historical factors in two risk assessment instruments in a group of violent offenders with schizophrenia. *Nord J Psychiatry* 2001; **55**: 243–9.

93 Tengstrom A, Grann M, Langstrom N, Kullgren G. Psychopathy (PCL-R) as a predictor of violent recidivism among criminal offenders with schizophrenia. *Law Hum Behav* 2000; **24**: 45–58.

94 Troquete NAC, Van Den Brink RHS, Beintema H, *et al.* Predictive validity of the short-term assessment of risk and treatability for violent behavior in outpatient forensic psychiatric patients. *Psychol Assess* 2015; **27(2)**: 377–91.

95 Thomson L, Davidson M, Brett C, Steele J, Darjee R. Risk assessment in forensic patients with schizophrenia: The predictive validity of actuarial scales and symptom severity for offending and violence over 8 - 10 years. *Int J Forensic Ment Health* 2008; **7**: 173–89.

96 van Heesch B, Jeandarme I, Pouls C, Vervaeke G. Validity and reliability of the VRAG in a forensic psychiatric medium security population in Flanders. *Psychol Crim Law* 2016; **22**: 530–7.

97 Vojt G, Thomson LD, Marshall LA. The predictive validity of the HCR-20 following clinical implementation: Does it work in practice? *J Forensic Psychiatry Psychol* 2013; **24**: 371–85.

98 Wallinius M, Nilsson T, Hofvander B, Anckarsater H, Stalenheim G. Facets of psychopathy among mentally disordered offenders: Clinical comorbidity patterns and prediction of violent and criminal behavior. *Psychiatry Res* 2012; **198**: 279–84.
